# Regioselective Alcoholysis of Silychristin Acetates Catalyzed by Lipases ‡

**DOI:** 10.3390/ijms160611983

**Published:** 2015-05-26

**Authors:** Eva Vavříková, Paolo Gavezzotti, Kateřina Purchartová, Kateřina Fuksová, David Biedermann, Marek Kuzma, Sergio Riva, Vladimír Křen

**Affiliations:** 1 Laboratory of Biotransformation, Institute of Microbiology, Academy of Sciences of the Czech Republic, Vídeňská 1083, CZ 142 20 Prague, Czech Republic; E-Mails: vavrikova@biomed.cas.cz (E.V.); katerina.purchartova@gmail.com (K.P.); karol.fuksova@gmail.com (K.F.); david.biedermann@gmail.com (D.B.); kuzma@biomed.cas.cz (M.K.); 2 Istituto di Chimica del Riconoscimento Molecolare, Consiglio Nazionale delle Ricerche, Via Mario Bianco 9, I 20131 Milano, Italy; E-Mail: paolo.gavezzotti@gmail.com

**Keywords:** silychristin, silymarin, lipase, acetylation, alcoholysis

## Abstract

A panel of lipases was screened for the selective acetylation and alcoholysis of silychristin and silychristin peracetate, respectively. Acetylation at primary alcoholic group (C-22) of silychristin was accomplished by lipase PS (*Pseudomonas cepacia*) immobilized on diatomite using vinyl acetate as an acetyl donor, whereas selective deacetylation of 22-*O*-acetyl silychristin was accomplished by Novozym 435 in methyl *tert*-butyl ether/*n*-butanol. Both of these reactions occurred without diastereomeric discrimination of silychristin A and B. Both of these enzymes were found to be capable to regioselective deacetylation of hexaacetyl silychristin to afford penta-, tetra- and tri-acetyl derivatives, which could be obtained as pure synthons for further selective modifications of the parent molecule.

## 1. Introduction

**Figure 1:**
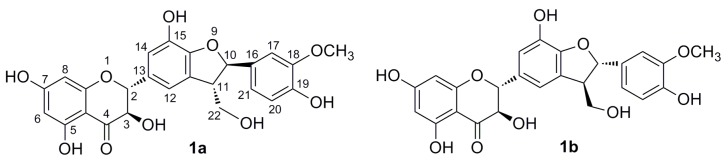
Structures of silychristin A (**1a**) and silychristin B (**1b**).

Silymarin [1], a crude extract from fruits of the milk thistle (*Silybum marianum*), contains flavonolignans occurring as pairs of diastereoisomers [2] (silybin A and B, isosilybin A and B, silychristin A and B) and other related compounds (silydianin, taxifolin; only single stereoisomers). This unique mixture of flavonolignans is largely used in a plethora of nutraceutics due to their hepatoprotective activity and also to other beneficial effects. So far, silybins A and B, accounting for *ca.* 30% of silymarin, are the only readily-available pure components from silymarin. The compounds in the remaining fraction of silymarin have only been isolated, up to very recently, in small quantities using laborious procedures, e.g., repetitive preparatory HPLC, and so, they could only be used for analytical standards. In 2014, a novel method for the preparatory separation of the minority silymarin components using Sephadex LH-20 gel chromatography was reported. This protocol gives access mainly to pure silydianin and silychristin (Figure 1) in multi-gram quantities [3].

The content of silychristin in milk thistle fruits varies according to the origin (region) where the milk thistle is grown and according to respective plant cultivars. Natural silychristin (**1**) exists as a mixture of two diastereoisomers: silychristin A (**1a**) and silychristin B (**1b**). Due to the high prevalence of silychristin A in silymarin [3], silychristin was initially considered to be a single diastereoisomer, with the absolute configuration 10*R*, 11*S* [4]. Later on, two diastereomeric forms of silychristin in a ratio 9:1 were observed [5], and in 2005, Smith and coworkers isolated a small amount of the second diastereoisomer of silychristin with the configuration 10*S*, 11*R*. Its structure was confirmed by NMR, and the compound was named silychristin B (**1b**) [6].

Silychristin has a number of beneficial properties, compared to the other silymarin flavonolignans. Its antioxidant and antiradical activity is twice as high as that of silybin [7]. This may be due to the higher polarity of this compound (six OH groups in the molecule), which is soluble in protic solvents (contrary to silybin) and, therefore, in principle, is more bioavailable (including water concoctions of *Silybum* fruits) [8].

The chemistry of flavonoids, similarly to other complex multifunctional natural products, is complicated by the need for complex protection/deprotection strategies due to the large number of chemo-equivalent groups. Further problems stem from the high sensitivity of flavonolignans towards oxidative agents and alkaline conditions and their tendency to complexate some metals. This limits the use of numerous protection/deprotection strategies. The application of enzymes may avoid these limitations, both in terms of selectivity and mild conditions [9].

Silychristin has been often discarded after the separation of silybin from silymarin, and as a consequence, its chemistry and biological activities have remained mostly unexplored. The pioneering chemical work on this molecule produced 3,5,7,15,19,22-hexa-*O*-acetyl silychristin, 3,22-di-*O*-acetyl-5,7,15,19-tetra-*O*-methyl silychristin or 5,7,15,19-tetra-*O*-methyl silychristin derivatives, whose structures were unequivocally determined [4]. More recently, 7-*O*-benzyl silychristin was prepared to investigate its laccase-catalyzed oxidation to obtain dimers [10].

Enzymatic protection/deprotection of primary OH groups has been reported to be useful in multi-step organic synthesis of flavonolignans. Acyl formations by lipases were used for the preparation of several derivatives of silymarin compounds, e.g., silybin [11]. For instance, acetylation at C-23 OH of silybin diastereomeric mixture and the following selective hydrolysis afforded optically pure diastereoisomers in multi-gram quantities [12,13,14].

In this work, the reaction protocols for the regioselective alcoholysis of silybin diastereoisomers catalyzed by lipase AK [14] have been extended to silychristin. Specifically, this paper presents the results obtained by screening a set of lipases for the enzymatic acetylation and alcoholysis at the C-22 OH group of silychristin. Subsequently, a basic kinetic study on the regioselective alcoholysis of peracetylated silychristin with the best performing lipases allowed the preparation of selectively-acetylated derivatives of silychristin suitable for further chemo-enzymatic modifications.

## 2. Results and Discussion

### 2.1. Enzymatic Acetylation of Silychristin (**1**)

This study deals with lipase-catalyzed reactions aimed at selectively modifying the molecule of silychristin. The idea of the preparation of 22-*O*-acetyl silychristin was inspired by our previous work engaged in the diastereomeric discrimination [13] of the mixture of silybin A and silybin B, which was accomplished by the combination of regioselective acetylation at C-23 OH and subsequent alcoholysis of 23-*O*-acetyl silybin.

**Scheme 1:**
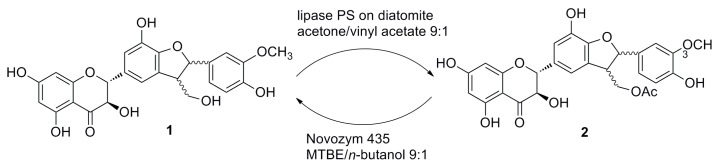
Enzymatic acetylation of silychristin (**1**) and alcoholysis of 22-*O*-acetyl-silychristin (**2**). Lipase PS (*Pseudomonas cepacia*), Sigma; MTBE, methyl *tert*-butyl ether.

**Figure 2:**
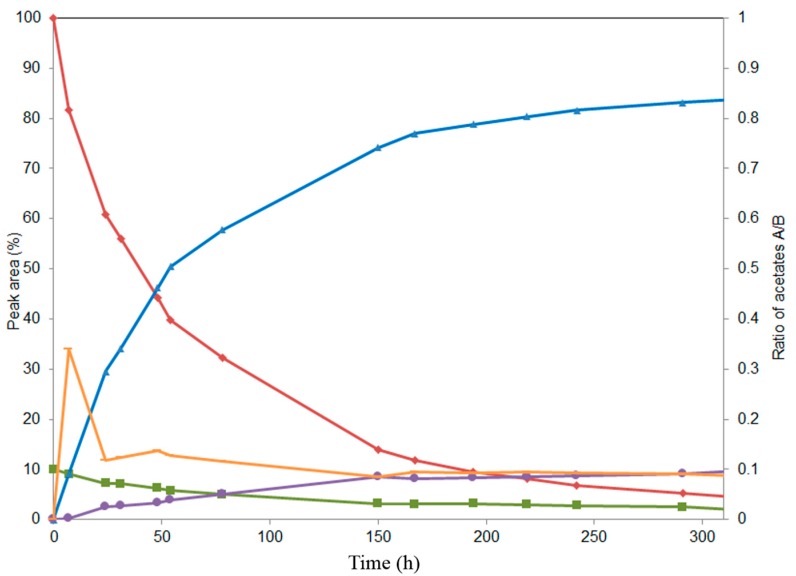
Time course study of lipase PS-catalyzed acetylation of natural silychristin composed of silychristin A (♦) and silychristin B (■) yielding 22-*O*-acetyl derivatives, A (▲) and B (●); (+) ratio of peak areas of 22-*O*-acetyl silychristin A:22-*O*-acetyl silychristin B (secondary axis *y*; please note that this axis has a different scale).

A panel of lipases were screened (Table 1), and various solvents (acetone, *tert*-amyl alcohol, toluene, dioxane, THF) were tested for the acetylation of natural silychristin (**1a**/**1b** = 9:1; we use hereunder the term “silychristin (**1**)” for this mixture if not otherwise stated). The best result was achieved with lipase PS (*Pseudomonas cepacia*) immobilized on diatomite in acetone in the presence of vinyl acetate as the acyl donor (Scheme 1). This enzymatic acetylation proceeded to give a quantitative yield of 22-*O*-acetyl silychristin without any traces of side products and allowed a simple reaction mixture workup. However, virtually no diastereomeric discrimination of silychristin A and B was observed during time course study (Figure 2).

**Table 1:** Screening of lipases for silychristin acetylation/deacetylation.

Enzyme	Source	Provider	Acetylation ^a^	Alcoholysis of 22-Acetate (2) ^b^	Alcoholysis of Peracetate (3) ^c^
Lipase AK	*Pseudomonas* sp*.*	*Amano*	NR ^d^	NR	NR
Lipase PS immobilized on celite	*Pseudomonas cepacia*	*Amano*	++	NR	++
Lipase PS immobilized on diatomite	*Pseudomonas cepacia*	*Sigma*	++	NR	++
PPL	porcine pancreas	*Sigma*	NR	NR	NR
Novozym 435	*Candida antarctica* (Lip. B)	*Novozymes*	+	++	++
Lipase G	*Penicillium camemberti*	*Amano*	NR	NR	NR
Lipase CE	*Humicola lanuginosa*	*Amano*	NR	NR	+
Lipase A	*Aspergillus niger*	*Amano*	NR	NR	+
Lipase F-AP15	*Rhizopus oryzae*	*Amano*	NR	NR	NR
Lipase CV	*Chromobacterium viscosum*	*Amano*	NR	NR	NR
Lipase CR	*Candida rugosa*	*Amano*	NR	NR	+
Lipozyme	*Rhizomucor miehei*	*Amano*	+	+	+
Lipolase	*Thermomyces lanuginosa*	*Amano*	+	+	+

^a^ Lipases tested for silychristin (**1**) acetylation. Reactions were terminated after 2 days; ^b^ Lipases tested for 22-*O*-acetyl silychristin (**2**) alcoholysis. Reactions were terminated after 4 days; ^c^ Lipases tested for 3,5,7,15,19,22-hexa-*O*-acetyl-silychristin (**3**) alcoholysis. Reactions were terminated after 2 days; ^d^ ++ conversion > 10%; + conversion < 10%; NR, no reaction.

### 2.2. Alcoholysis of 22-O-Acetyl Silychristin (**2**)

**Figure 3:**
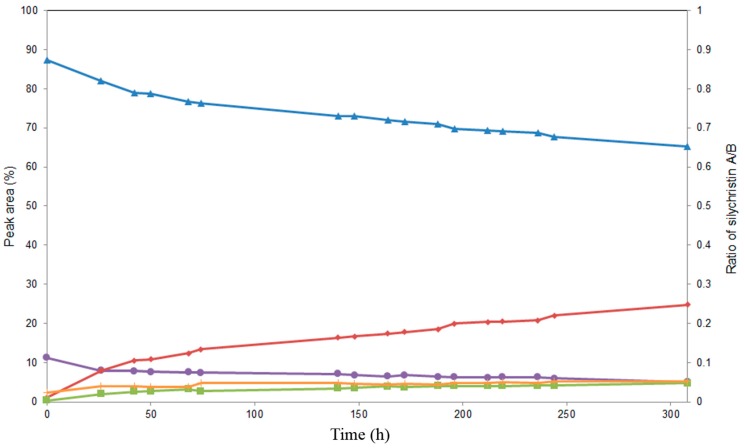
Time course study of Novozym 435 catalyzed deacetylation of 22-*O*-acetyl silychristins A (▲) and B (●) yielding the corresponding silychristin A (♦) and silychristin B (■); (+) ratio of peak areas of silychristin A:silychristin B (secondary axis *y*; please note that this axis has a different scale).

The alcoholysis of 22-*O*-acetyl silychristin (**2**) was attempted to achieve silychristin diastereomeric discrimination (Scheme 1). Alcoholysis in various solvents (MTBE, *tert*-amyl alcohol and toluene), previously found to be suitable for the discrimination of 22-*O*-acetyl silybin, was tested [14]. Among the two previously successful enzymes—Novozym 435 and lipase PS immobilized on diatomite—the only positive result was obtained with Novozym 435 in a mixture of solvents MTBE and *n*-butanol. The course of the reaction was monitored by HPLC, evaluating the formation of the diastereoisomers, **1a** and **1b** (Figure 3). Once again, no diastereomeric discrimination good enough for the kinetic resolution of the mixture of 22-*O*-acetyl silychristin A and B (**2**) was observed.

### 2.3. Screening of Lipases for the Selective Alcoholysis of 3,5,7,15,19,22-Hexa-O-acetyl silychristin (**3**)

The goal of this set of experiments was to find an efficient method for the preparation of selectively protected silychristin synthons, suitable for further derivatization, from the easily available silychristin peracetate (**3**).

>The regioselective actions of various lipases in the deacetylation of several flavonoids have been described in the literature. For instance, Lambusta *et al*. [15] described the selective deacetylation of quercetin by two different lipases. Novozym 435 removed the C-4′ acetate [15,16] from quercetin peracetate, while Lipozyme [16] or lipase from *Mucor miehei* [15] preferred the alcoholysis of the acetyl group at C-7. An analogous study was performed with the peracetylated morin, structurally similar to quercetin. The C-4′ position was deprotected by Novozym 435 after a long reaction time, then the deacetylation progressed at the C-7 OH. On the other hand, Lipozyme recognized substituents on the A and B rings of peracetyl morin, and the 3,5,2′-tri-*O*-acetyl morin was isolated as the final product [16]. In another study [17], fully acetylated luteolin, kaempferol, kaempferide and quercetin were exposed to alcoholysis catalyzed by lipase from *P. cepacia*, which yielded various products, e.g., 5,7,4′-tri-*O*-acetyl luteolin and 5,7-di-*O*-acetyl luteolin. Peracetylated quercetin was converted into 3,5,3′,4′-tetra-*O*-acetyl quercetin, 3,5,3′-tri-*O*-acetyl quercetin, 3,3′,4′-tri-*O*-acetyl quercetin and 3-*O*-acetyl quercetin. More recently, pentaacetyl silybins A and/or B were prepared [14]. From the many lipases tested, only lipase AK provided good conversion during the deacetylation of silybins. Reactions yielded at the beginning 3,5,20,23-tetra-*O*-acetyl silybins A and/or B and then 3,20,23-tri-*O*-acetyl silybins A and/or B. Reactions were tested on the diastereoisomeric mixtures, as well as on optically pure silybins, which always yielded the same types of products.

In this work, thirteen lipases were tested for the selective alcoholysis of **3**, and three of them gave conversion >10% (Table 1). Sufficient conversion rates were observed with Novozym 435, lipase PS immobilized on celite and lipase PS immobilized on diatomite. The two lipase PS preparations gave a similar yield; thus, for large-scale reactions, only the commercially available lipase PS on diatomite and the well-known Novozym 435 were used. Once again, no diastereomeric discrimination good enough for kinetic resolution between silychristin A and silychristin B derivatives was observed.

### 2.4. Alcoholysis of 3,5,7,15,19,22-Hexa-O-acetyl silychristin (**3**)

**Scheme 2:**
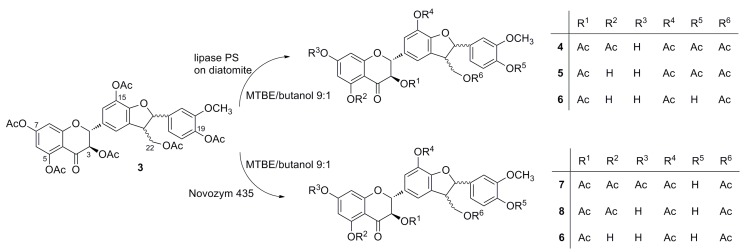
Regioselective alcoholysis of silychristin peracetate (**3**) catalyzed by lipase PS on diatomite or by Novozym 435.

**Figure 4:**
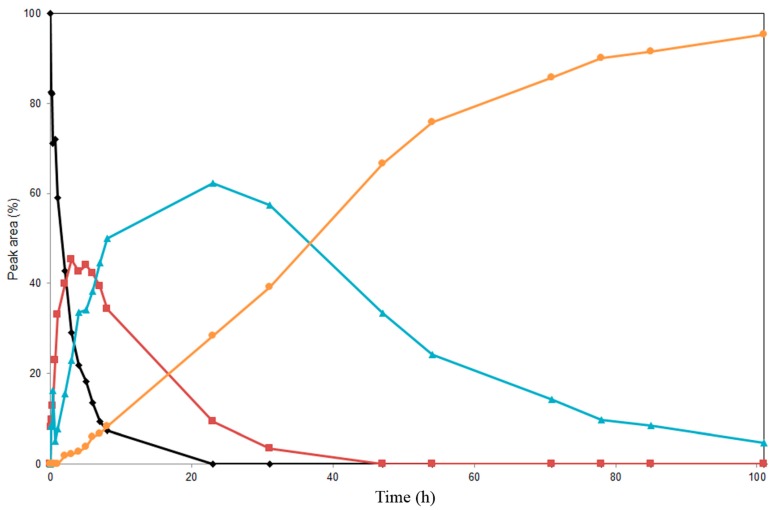
Time course study of the alcoholysis of silychristin peracetate (**3**, ♦) at 45 °C in MTBE/*n*-butanol catalyzed by lipase PS immobilized on diatomite. Products (3,5,15,19,22-penta-*O*-acetyl silychristin, **4**, ■), (3,15,19,22-tetra-*O*-acetyl silychristin, **5**, ▲) and (3,15,22-tri-*O*-acetyl silychristin, **6**, ●).

**Figure 5:**
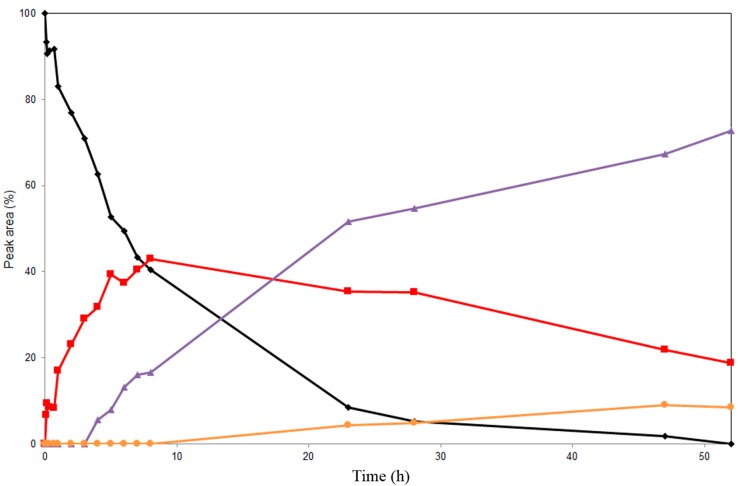
Time course study of the alcoholysis of silychristin peracetate (**3**, ♦) at 45 °C in MTBE/*n*‑butanol catalyzed by Novozym 435. Products (3,5,7,15,22-penta-*O*-acetyl silychristin, **7**, ■), (3,5,15,22-tetra-*O*-acetyl silychristin, **8**, ▲) and (**6**, ●).

Based on the above screening, Novozym 435 and lipase PS immobilized on diatomite were used for preparatory-scale alcoholysis experiments. Product formation during the regioselective alcoholysis of **3** was monitored by HPLC (Figure 4 and Figure 5). The reactions catalyzed by Novozym 435 and by lipase PS immobilized on diatomite afforded different products (Scheme 2). All of the compounds were isolated and characterized by mass spectrometry and complex NMR investigation, allowing their unequivocal structural determination. Control experiments proved that no spontaneous alcoholysis of **3** occurred in the absence of the enzymes.

In the course of alcoholysis catalyzed by lipase PS immobilized on diatomite (Figure 4), the C-7 acetate was removed first, yielding 3,5,15,19,22-penta-*O*-acetyl silychristin (**4**, MS-ESI *m*/*z*: [M + Na]^+^ 715.1 Da); subsequent C-5 acetate removal afforded 3,15,19,22-tetra-*O*-acetyl silychristin (**5**, MS-ESI *m*/*z*: [M + Na]^+^ 673.2 Da); and finally, C-19 acetate removal yielded 3,15,22-tri-*O*-acetyl silychristin (**6**, MS-ESI *m*/*z*: [M + Na]^+^ 631.0 Da) in a reasonable purity (95%) after 100 hours. Thus, selectively-deacetylated products could be obtained during the distinct time course of the reaction: Hour 6, pentaacetate **4**; Hour 23, tetraacetate **5**; Hour 100, triacetate **6**. A TLC of a reaction catalyzed by lipase PS is reported in the Figure S3. Products **5** and **6** could be isolated in pure form, whereas compound **4** was always isolated as a mixture with the tetraacetate silychristin **5**.

The alcoholysis of **3** catalyzed by Novozym 435 went through a different pathway (Figure 5); in the first step, the C-19 acetate was cleaved to produce 3,5,7,15,22-penta-*O*-acetyl silychristin (**7**, MS-ESI *m*/*z*: [M + Na]^+^ 715.6 Da), followed by the removal of the C-7 acetate to give 3,5,15,22-tetra-*O*-acetyl silychristin (**8**, MS-ESI *m*/*z*: [M + Na]^+^ 673.2 Da) in a 73% yield after 52 h. The structure of the third derivative was found to be identical to the previously isolated compound **6** (3,15,22-tri-*O*-acetyl silychristin) obtained with lipase PS. Due to the considerably lower yield (18.3%) of **6** after 52 h with Novozym 435, the use of lipase PS for the production of this compound is more advantageous (Figure 4). A TLC of the reaction catalyzed by Novozym 435 is also reported in Figure S4. The three compounds, **6**, **7** and **8**, could be isolated in pure form.

Unfortunately, not all OH signals were visible in the ^1^H NMR spectra of the isolated acetyl silychristin derivatives in DMSO-*d*_6_. Therefore, it was impossible to use the presence of the signals of some of them (compared to the parent compound) as a proof of the deacetylation site. To overcome this, the four-bond heteronuclear correlation between methyl protons of the acetyl group and the acetylated carbon was used as direct evidence for its substitution. Moreover, the heteronuclear coupling between H-3 or H-22 and the CH_3_COO of acetyl was used as a direct proof of the acetylation at these positions.

Having the kinetic information in hand, it is now possible to terminate the alcoholysis reactions at specific times to obtain penta-, tetra- or tri-acetyl derivatives of silychristin in good yields.

## 3. Experimental Section

### 3.1. Chemicals and Reagents

Silymarin was purchased from Liaoning Senrong Pharmaceutical Co., Ltd. (Liaoning, China). Silychristin (**1**) was isolated from silymarin by gel filtration [3] using Sephadex LH-20. Silychristin (**1**) was used as the mixture of two diastereomeric forms A and B in a ratio 9:1. Lipase B from *Candida antarctica* immobilized on acrylic resin (Novozym 435) was purchased from Novo-Nordisk (Copenhagen, Denmark). Amano Lipase PS immobilized on diatomite, as well as other chemicals were obtained from Sigma-Aldrich (Prague, Czech Republic). Lipase PS immobilized on celite was prepared from lipase from *P. cepacia* (Amano, Nagoya, Japan) and celite (Hyflo^®^ Super Cell, Sigma–Aldrich, Prague, Czech Republic) in 0.1 M phosphate buffer (pH 7). Lipase PS, lipase G, lipase CE, lipase A, lipase F-AP15, lipase CV, lipase CR, Lipozym and Lipolase were obtained from Amano. Porcine pancreas lipasePPL was obtained from Sigma–Aldrich (Prague, Czech Republic).

### 3.2. General Methods

NMR spectra were recorded on a Bruker Avance III 700 MHz spectrometer (700.13 MHz for ^1^H, 176.05 MHz for ^13^C at 30 °C) (Bruker Daltonik, Bremen, Germany) and a Bruker Avance III 600 MHz spectrometer (600.23 MHz for ^1^H, 150.93 MHz for ^13^C at 30 °C) in DMSO-*d*_6_ (99.8% atom D, ARMAR Chemicals, Dӧttingen, Switzerland). The residual signal of the solvent was used as an internal standard (δ_H_ 2.500 ppm, δ_C_ 39.60 ppm). NMR experiments—^1^H NMR, ^13^C NMR, COSY, HSQC, HMBC and 1D TOCSY—were performed using the manufacturer’s software. ^1^H NMR and ^13^C NMR spectra were zero filled to four-fold data points and multiplied by a window function before Fourier transformation. The two-parameter double-exponential Lorentz–Gauss function was applied for ^1^H to improve resolution, and line broadening (1 Hz) was applied to get a better ^13^C signal-to-noise ratio. Chemical shifts are given in δ-scale with digital resolution justifying the reported values to three (δ_H_) or two (δ_C_) decimal places.

Mass spectra were recorded on a Micromass Platform LC system (Waters, Milford, MA, USA) in methanol with the addition of formic acid.

### 3.3. HPLC

Method A: For acetylation/deacetylation of silychristin (**1**) and 22-*O*-acetyl silychristin (**2**), the following HPLC system was used: Twin Watrex (Prague, Czech Republic) Deltachrom SDS (030) pumps equipped with Thermo Separation Spectra 100 UV/V detector; RP-18e Chromolith ^®^ SpeedRod (50 mm × 4.6 mm, Merck, Darmstadt, Germany); Solvent System A: MeOH/H_2_O/HCO_2_H (20/80/0.1, *v*/*v*/*v*); and B: MeOH/HCO_2_H (100/0.1, *v*/*v*); gradient: 0–4 min 100% A, 4–8 min 100%–50% A, 8–9 min 50% A, 9–10 min 50%–100% A, 10–11 min 100% A; flow rate 1.1 mL/min; the signal at 285 nm was acquired.

Method B: For analytical evaluation of partially-acetylated silychristins (**3**–**8**) (Figure 4 and Figure 5), the following HPLC system was used: Jasco 880-PU (Jasco Europe, Cremella, Italy) pump equipped with a Jasco 875-UV/V detector; RP-18e (5 μm) column (Purosphere STAR, 100 mm × 3 mm i.d., Merck, Darmstadt, Germany); Solvent System A: MeCN/H_2_O/HCO_2_H (5/95/0.1, *v*/*v*/*v*); and B: MeCN/HCO_2_H (100/0.1, *v*/*v*). The optimal gradient for monitoring the reaction catalyzed by Novozym 435 was 0–1 min 40% B, 1–20 min 40%–70% B, 20–22 min 70% B, 22–24 min 40% B. The optimal gradient for monitoring the reaction catalyzed by lipase PS was 0–1 min 30% B, 1–20 min 30%–80% B, 20–22 min 80% B, 22–24 min 30% B. The flow rate was 1.1 mL/min. The data were acquired at 285 nm.

Method C: HPLC analysis of the inseparable mixture of two products, 3,5,15,19,22-penta-*O*-acetyl-silychristin (**4**) and 3,15,19,22-tetra-*O*-acetyl silychristin (**5**), was performed on Twin Watrex (Prague, Czech Republic) Deltachrom SDS (030) pumps equipped with Thermo Separation Spectra 100 UV/V detector; RP-18e Chromolith^®^ SpeedRod (50 mm × 4.6 mm, Merck, Darmstadt, Germany); Solvent System A: MeOH/H_2_O/HCO_2_H (20/80/0.1, *v*/*v*/*v*); and B: MeOH/HCO_2_H (100/0.1, *v*/*v*). Gradient: 0–2 min 100% A, 2–3 min 100%–60% A, 3–5 min 60%–20% A, 5–10 min 20%–60% A, 10–17 min 60%–100% A; flow rate 0.9 mL/min; the signal at 285 nm was acquired.

### 3.4. Chemistry

#### 3.4.1. Screening of Enzymatic Acetylation of Silychristin

Silychristin (**1**, 5 mg, 18.9 μmol) was dissolved in a mixture of acetone/vinyl acetate (1 mL, 9:1, *v*/*v*) in a vessel containing the respective lipase (Novozym 435, Lipolase 15 mg; lipase PPL, lipase CR, Lipozyme, lipase AK, lipase CE, lipase G, lipase A, lipase F-AP15 each 25 mg; lipase PS, lipase CV each 50 mg). The reaction mixtures were incubated at 45 °C and 600 rpm in a Thermomixer (Eppendorf, Hamburg, Germany); the reactions progress was monitored by TLC (mobile phase: CHCl_3_/toluene/acetone/HCO_2_H, 12:2:2:0.1) and by HPLC (Method A).

#### 3.4.2. Enzymatic Synthesis of 22-*O*-Acetyl-silychristin (**2**)

Lipase PS immobilized on diatomite (375 mg) was added to a solution of silychristin (**1**, 75 mg) in acetone/vinyl acetate (20 mL, 9:1, *v*/*v*), and the mixture was shaken at 45 °C and 650 rpm in a Thermomixer (Eppendorf, Hamburg, Germany). After 300 hours, the reaction was terminated by filtering off the enzyme, and the solvent was evaporated under reduced pressure. The crude product **2** was purified by silica gel flash chromatography (CHCl_3_/toluene/acetone, 50:45:5–40:35:25) to afford the product as a yellowish solid (yield 70%, MS-ESI *m*/*z*: [M + Na]^+^ calculated for C_27_H_24_O_11_Na 547.1; found 547.0). For ^1^H and ^13^C NMR data, see Tables S1 and S2.

#### 3.4.3. Enzymatic Alcoholysis of 22-*O*-Acetyl-silychristin (**2**)

Novozym 435 (75 mg) was added to a solution of 22-*O*-acetyl-silychristin (**2**, 75 mg) in methyl *tert*-butyl ether (MTBE)/*n*-butanol (4 mL, 9:1, *v*/*v*), and the mixture was shaken at 45 °C and 650 rpm. After 300 h, the reaction was terminated by filtering off the enzyme, and the solvent was evaporated under reduced pressure. The crude product **1** was purified by silica gel flash chromatography (CHCl_3_/toluene/acetone 50/45/5–40/35/25) to afford the product as a white solid (yield 55%).

#### 3.4.4. Preparation of 3,5,7,15,19,22-Hexa-*O*-acetyl-silychristin (**3**)

Natural silychristin (silychristin A (**1a**) and silychristin B (**1b**) in a ratio 9:1; **1**, 150 mg, 0.31 mmol) was peracetylated with a mixture of Ac_2_O in EtOAc (25 mL, EtOAc/Ac_2_O, 25:0.6, *v*/*v*), 4-(dimethylamino)-pyridine (3 mg, 24.5 μmol) and triethylamine (600 μL). The reaction mixture was stirred at room temperature overnight, then evaporated and purified by flash chromatography on silica gel (CHCl_3_/toluene/acetone 70:25:5) yielding the title compound (**3**) as a white amorphous solid (120 mg, 52.6%), MS-ESI *m*/*z*: [M + Na]^+^ calculated for C_37_H_34_O_16_Na 757.2; found 757.7. For ^1^H and ^13^C NMR data, see Tables S1 and S2, Figures S8 and S9.

#### 3.4.5. Screening of Enzymatic Alcoholysis of 3,5,7,15,19,22-Hexa-*O*-acetyl-silychristin (**3**)

The screening of lipases for the alcoholysis of silychristin peracetate (**3**, 3 mg, 4.1 μmol) was accomplished in the solution of MTBE/*n*-butanol (1.8 mL, 9:1, *v*/*v*) containing the respective lipase (50 mg for various lipases or 10 mg for Novozym 435). The reaction mixtures were incubated at 45 °C and 600 rpm in a Thermomixer (Eppendorf, Hamburg, Germany); the reaction progresses was monitored by TLC (mobile phase: CHCl_3_/toluene/acetone/HCO_2_H 12:2:2:0.1) and by analytical HPLC.

#### 3.4.6. Preparative Alcoholysis of 3,5,7,15,19,22-Hexa-*O*-acetyl silychristin (**3**) Catalyzed by Lipase PS on Diatomite

3,5,7,15,19,22-Hexa-*O*-acetyl silychristin (**3**, 160 mg, 0.22 mmol) was dissolved in a mixture of MTBE and *n*-butanol (90 ml, 9:1, *v*/*v*) with the addition of lipase PS immobilized on diatomite (1 g plus 1 g after 31 hours of reaction). The reaction mixture was incubated at 45 °C under shaking (200 rpm) for 4 days, and the reaction progress was monitored by TLC (mobile phase: CHCl_3_/toluene/acetone/HCO_2_H, 12/2/2/0.1). Aliquots of the reaction mixture were taken and dried for monitoring the reaction progress by HPLC (Method B). The residue, after removing the enzyme and evaporation of the solvent, was purified by flash chromatography on silica gel (mobile phase: CHCl_3_/toluene/acetone in gradient 75/20/5–80/10/10) yielding the minor product 3,15,19,22-tetra-*O*-acetyl silychristin (**5**, 6 mg), 4.2%, MS-ESI *m*/*z*: [M + Na]^+^ calcd. for C_33_H_30_O_14_Na 673.2; found 673.2; and the major product 3,15,22-tri-*O*-acetyl silychristin (**6**, 122 mg), 91.2%, MS-ESI *m*/*z*: [M + Na]^+^ calculated for C_31_H_28_O_13_Na 631.1; found 631.0; all obtained as white amorphous solids. For ^1^H and ^13^C NMR data for **5**, **6**, see Tables S1 and S2, Figures S10–S13. For the HPLC chromatogram, see Figure S1.

#### 3.4.7. Preparation of 3,5,15,19,22-Penta-*O*-acetyl-silychristin (**4**) Catalyzed by Lipase PS on Diatomite

3,5,7,15,19,22-Hexa-*O*-acetyl-silychristin (**3**, 50 mg, 0.07 mmol) was dissolved in a mixture of MTBE and *n*-butanol (40 mL, 9:1, *v*/*v*) with the addition of lipase PS immobilized on diatomite (400 mg). The reaction mixture was incubated at 45 °C under shaking (200 rpm) for 6 h, and the reaction progress was monitored by TLC (mobile phase: CHCl_3_/toluene/acetone/HCO_2_H, 12/2/2/0.1). The reaction mixture after removing the enzyme and solvent evaporation was separated on LH 20 Sephadex^®^ in 100% acetone affording the mixture (inseparable) of two products 3,5,15,19,22-penta-*O*-acetyl-silychristin (**4**) and 3,15,19,22-tetra-*O*-acetyl silychristin (**5**) in a ratio 2:1, which were characterized by MS: **4**, MS-ESI *m*/*z*: [M + Na]^+^ calculated for C_35_H_32_O_15_Na 715.2; found 715.1; **5**, MS-ESI *m*/*z*: [M + Na]^+^ calculated for C_33_H_30_O_14_Na 673.2; found 673.0; and by NMR. For ^1^H and ^13^C NMR data for **4**, see Tables S1 and S2, Figures S6 and S7. The mixture was analyzed by HPLC (Method C); see Figure S5.

#### 3.4.8. Preparative Alcoholysis of 3,5,7,15,19,22-Hexa-*O*-acetyl silychristin (**3**) catalyzed by Novozym 435

3,5,7,15,19,22-Hexa-*O*-acetyl silychristin (**3**, 200 mg, 0.27 mmol) was dissolved in a mixture of MTBE and *n*-butanol (120 ml, 9:1, *v*/*v*) with the addition of lipase Novozym 435 (550 mg). The reaction mixture was incubated at 45 °C under shaking (200 rpm) for 52 h, and the reaction progress was monitored by TLC (mobile phase: CHCl_3_/toluene/acetone/HCO_2_H, 12/2/2/0.1). Aliquots of the reaction mixture were taken and dried for monitoring the reaction progress by HPLC. The residue, after removing the enzyme and evaporation of the solvent, was purified by flash chromatography on silica gel (mobile phase: CHCl_3_/toluene/acetone in gradient 75/20/5–80:10:10) yielding three products: 3,5,7,15,22-penta-*O*-acetyl silychristin (**7**, 58 mg), 31.0%, MS-ESI *m*/*z*: [M + Na]^+^ calcd. for C_35_H_32_O_15_Na 715.2; found 715.6; 3,5,15,22-tetra-*O*-acetyl silychristin (**8**, 73 mg), 41.6%, MS-ESI *m*/*z*: [M + Na]^+^ calcd. for C_33_H_30_O_14_Na 673.2; found 673.2; and 3,15,22-tri-*O*-acetyl silychristin (**6**, 30 mg), 18.3%, MS-ESI *m*/*z*: [M + Na]^+^ calcd. for C_31_H_28_O_13_Na 631.1; found 631.0; all obtained as white amorphous solids. For ^1^H and ^13^C NMR data for **7**, **8**, see Tables S1 and S2, Figures S14–S17. For the HPLC chromatogram, see Figure S2.

## 4. Conclusions

A panel of lipases was screened for the selective acetylation and alcoholysis of silychristin and silychristin peracetate, respectively. Acetylation at the primary alcoholic group (C-22) of silychristin was accomplished by lipase PS immobilized on diatomite using vinyl acetate in acetone as an acetyl donor. Deacetylation of 22-*O*-acetyl silychristin (**2**) could be accomplished by Novozym 435 in MTBE/*n*-butanol. However, in both of these protocols, no diastereomeric discrimination good enough for the kinetic resolution of silychristin A and B was observed, and therefore, this approach cannot be used for the separation of the silychristin isomers. Both lipase PS immobilized on diatomite and Novozym 435 were found to catalyze the regioselective deacetylation of hexaacetyl silychristin to afford, in a good yield, 3,15,19,22-tetra-*O*-acetyl silychristin (**5**), 3,15,22-tri-*O*-acetyl silychristin (**6**), 3,5,7,15,22-penta-*O*-acetyl silychristin (**7**) and 3,5,15,22-tetra-*O*-acetyl silychristin (**8**). These compounds, as well as the silychristin monoacetate (**2**), can be used as pure synthons for further selective modifications of this valuable natural compound.

## Supplementary Materials

Supplementary materials can be found at http://www.mdpi.com/1422-0067/16/06/11983/s1.
