# Supplementary material for: Regioselective Alcoholysis of Silychristin Acetates Catalyzed by Lipases
Source: Int J Mol Sci. 2015 May 26;16(6):11983–95. doi: 10.3390/ijms160611983 (PMC4490424; doi:10.3390/ijms160611983)
Supplement: Supplementary file 1 [file ijms-16-11983-s001.pdf]

Supplementary Information

Table S1. ^{13}C NMR data of prepared compounds (DMSO-d_6, 30 °C).

Atom #	2		3		4		5		6		7		8	
	δ_C	m#	δ_C	m#	δ_C	m#	δ_C	m#	δ_C	m#	δ_C	m#	δ_C	m#
**2**	83.12	d	79.81	d	79.52	d	79.52	d	79.64	d	79.86	d	79.59	d
**3**	71.73	d	72.82	d	72.78	d	71.99	d	72.00	d	72.82	d	72.78	d
**4**	197.53	s	185.69	s	184.44	s	190.83	s	191.32	s	185.71	s	184.50	s
**4a**	100.34	s	110.46	s	105.19	s	100.32	s	100.64	s	110.46	s	105.28	s
**5**	163.36	s	150.81	s	151.92	s	163.30	s	163.28	s	150.81	s	151.92	s
**6**	96.17	d	111.51	d	105.84	d	96.79	d	96.60	d	111.50	d	105.80	d
**7**	167.28	s	156.27	s	165.06	–	168.2 *	–	167.40	s	156.27	s	164.91	s
**8**	95.15	d	109.25	d	101.04	d	95.76	d	95.53	d	109.25	d	101.04	d
**8a**	162.53	s	162.25	s	163.12	s	162.19	s	162.29	s	162.27	s	163.13	s
**10**	87.49	d	87.68	d	87.64	d	87.69	d	88.47	d	88.46	d	88.42	d
**11**	49.56	d	49.72	d	49.76	d	49.74	d	49.47	d	49.43	d	49.48	d
**11a**	127.76	s	129.29	s	129.18	s	129.22	s	129.66	s	129.71	s	129.61	s
**12**	115.10	d	122.21	d	122.03	d	122.03	d	121.91	d	122.07	d	121.90	d
**13**	130.47	s	128.72	s	129.12	s	129.11	s	128.66	s	128.37	s	128.76	s
**14**	116.24	d	123.07	d	122.95	d	122.89	d	122.84	d	123.00	d	122.88	d
**15**	140.93	s	133.10	s	133.06	s	133.09	s	133.08	s	133.07	s	133.04	s
**15a**	147.06	s	151.15	s	151.01	s	151.02	s	151.12	s	151.21	s	151.09	s
**16**	131.46	s	139.37	s	139.43	s	139.42	s	131.06	s	131.01	s	131.07	s
**17**	110.62	d	110.31	d	110.29	d	110.31	d	110.27	d	110.27	d	110.25	d
**18**	147.65	s	150.97	s	150.97	s	150.97	s	147.70	s	147.69	s	147.69	s
**19**	146.69	s	139.18	s	139.17	s	139.18	s	146.82	s	146.82	s	146.80	s
**20**	115.37	d	123.07	d	123.07	d	123.08	d	115.45	d	115.43	d	115.44	d
**21**	119.08	d	117.83	d	117.79	d	117.82	d	118.74	d	118.75	d	118.71	d
**22**	65.11	t	64.87	t	64.90	t	64.90	t	64.77	t	64.74	t	64.76	t
**18-OMe**	55.72	q	55.81	q	55.81	q	55.82	q	55.64	q	55.63	q	55.63	q
**3-CO**	–	–	168.54	s	168.62	s	168.74	s	168.73	s	168.55	s	168.63	s

Table S1. *Cont.*

Atom #	2		3		4		5		6		7		8	
	δ_C	m#	δ_C	m#	δ_C	m#	δ_C	m#	δ_C	m#	δ_C	m#	δ_C	m#
**5-CO**	–	–	168.53	s	168.55	–	–	–	–	–	168.53	s	168.55	S
**7-CO**	–	–	168.21	s	-	–	–	–	–	–	168.21	s	–	–
**15-CO**	–	–	168.26	s	168.26	s	168.28	s	168.24	s	168.21	s	168.22	s
**19-CO**	–	–	168.53	s	168.52	s	168.54	s	–	–	–	–	–	–
**22-CO**	170.39	s	170.31	s	170.31	s	170.33	s	170.33	s	170.32	s	170.32	s
**3-Ac**	–	–	20.05	q	20.11	q	20.11	q	20.09	q	20.06	q	20.12	q
**5-Ac**	–	–	20.73	q	20.85	q	–	–	–	–	20.73	q	20.85	q
**7-Ac**	–	–	20.91	q	–	–	–	–	–	–	20.91	q	–	–
**15-Ac**	–	–	20.35	q	20.35	q	20.37	q	20.38	q	20.36	q	20.37	q
**19-Ac**	–	–	20.40	q	20.40	q	20.41	q	–	–	–	–	–	–
**22-Ac**	20.62	q	20.52	q	20.51	q	20.54	q	20.53	q	20.52	q	20.52	q

* HMBC (Heteronuclear Multiple Bond Correlation) readout.

Table S2. ^{1}H NMR data of prepared compounds (DMSO-d_6, 30 °C).

Atom #	2			3			4			5			6			7			8		
	δ_H	m[#]	J_{H-H} [Hz]	δ_H	m[#]	J_{H-H} [Hz]	δ_H	m[#]	J_{H-H} [Hz]	δ_H	m[#]	J_{H-H} [Hz]	δ_H	m[##]	J_{H-H} [Hz]	δ_H	m[#]	J_{H-H} [Hz]	δ_H	m[#]	J_{H-H} [Hz]
**2**	5.020	d	11.2	5.720	d	12.4	5.544	d	12.1	5.546	d	11.8	5.560	d	11.9	5.714	d	12.4	5.540	d	12.1
**3**	4.517	dd	6.1, 11.2	5.971	d	12.4	5.757	d	11.1	5.924	d	11.8	5.943	d	11.9	5.968	d	12.4	5.757	d	12.1
**6**	5.896	d	2.0	6.790	d	2.2	6.259	d	2.2	5.922	d	2.0	5.967	d	2.1	6.786	d	2.2	6.265	d	2.2
**8**	5.848	d	2.0	6.904	d	2.2	6.314	d	2.2	5.901	d	2.0	5.944	d	2.1	6.907	d	2.2	6.322	d	2.2
**10**	5.439	d	7.7	5.717	d	6.6	5.714	d	6.4	5.715	d	6.4	5.571	d	6.8	5.568	d	6.9	5.566	d	6.8
**11**	3.764	dddd	0.6, 5.8, 7.4, 7.7	3.844	dddd	0.9, 5.9, 6.6, 6.7	3.843	m	–	3.844	dddd	0.8, 5.7, 6.4, 6.9	3.809	dddd	0.7, 5.8, 6.7, 6.8	3.808	dddd	0.8, 6.1, 6.8, 6.9	3.801	dddd	0.8, 6.0, 6.6, 6.8
**12**	6.892	dd	0.6, 1.7	7.475	dd	0.9, 1.7	7.442	dd	0.6, 1.6	7.424	dd	0.8, 1.7	7.408	dd	0.7, 1.6	7.453	dd	0.8, 1.7	7.420	dd	0.8, 1.7
**14**	6.865	d	1.7	7.272	d	1.7	7.244	d	1.6	7.228	d	1.7	7.212	d	1.6	7.249	d	1.7	7.221	d	1.7
**17**	6.984	d	2.0	7.159	d	2.0	7.156	d	1.9	7.155	d	2.0	6.949	d	1.8	6.950	d	1.9	6.947	d	1.8
**20**	6.780	d	8.1	7.105	d	8.2	7.105	d	8.2	7.105	d	8.2	6.772	d	8.0	6.770	d	8.0	6.770	d	8.1
**21**	6.823	dd	2.0, 8.1	6.970	dd	2.0. 8.2	6.967	dd	1.9, 8.2	6.967	dd	2.0. 8.2	6.793	dd	1.8. 8.0	6.794	dd	2.0. 8.0	6.790	dd	1.8. 8.1
**22α**	4.375	dd	5.8, 11.0	4.371	dd	5.9, 11.0	4.380	dd	5.6, 11.2	4.377	dd	5.7, 11.1	4.337	dd	5.8, 11.0	4.327	dd	6.1, 11.2	4.327	dd	6.0, 11.1
**22β**	4.249	dd	7.4, 11.0	4.344	dd	6.7, 11.0	4.347	dd	7.0, 11.2	4.345	dd	6.9, 11.1	4.311	dd	6.7, 11.0	4.304	dd	6.8, 11.2	4.305	dd	6.6, 11.1
**3-OH**	5.744	d	6.1	–	–	–	–	-	–	–	–	–	–	–	–	–	–	–	–	–	–
**5-OH**	11.898	s	–	–	–	–	–	-	–	11.451	s	–	11.426	s	–	–			–		
**7-OH**	10.859	br.s.	–	–	–	–	11.119	br.s.	–	n.d.	–	–	11.021	br.s.	–	–			11.129	br.s.	–
**15-OH**	9.412	s	–	–	–	–	–	-	–	–	–	–	–	–	–	-	–	–	–	–	–
**18-OMe**	3.769	s	–	3.768	s	–	3.769	s	–	3.768	s	–	3.752	s	–	3.750	s	–	3.751	s	–

Table S2. *Cont.*

Atom #	2			3			4			5			6			7			8		
	**δ_H**	**m#**	**$J_{H\text{-}H}$ [Hz]**	**δ_H**	**m#**	**$J_{H\text{-}H}$ [Hz]**	**δ_H**	**m#**	**$J_{H\text{-}H}$ [Hz]**	**δ_H**	**m#**	**$J_{H\text{-}H}$ [Hz]**	**δ_H**	**m##**	**$J_{H\text{-}H}$ [Hz]**	**δ_H**	**m#**	**$J_{H\text{-}H}$ [Hz]**	**δ_H**	**m#**	**$J_{H\text{-}H}$ [Hz]**
**19-OH**	9.047	s	–	–	–	–	-			–			9.064	s	–	9.064	s	–	9.061	s	–
**3-Ac**	–	–	–	1.942	s	–	1.927	s	–	1.954	s	–	1.963	s	–	1.948	s	–	1.933	s	–
**5-Ac**	–	–	–	2.300	s	–	2.260	s	–	–			–			2.300	s	–	2.261	s	–
**7-Ac**	–	–	–	2.281	s	–	–			-			–			2.282	s	–	–		
**15-Ac**	–	–	–	2.293	s	–	2.292	s	–	2.292	s	–	2.272	s	–	2.272	s	–	2.270	s	–
**19-Ac**	–	–	–	2.252	s	–	2.252	s	–	2.251	s	–	–			–			–		
**22-AC**	1.974	s	–	1.993	s	–	1.992	s	–	1.996	s	–	1.983	s	-	1.978	s	–	1.978	s	–

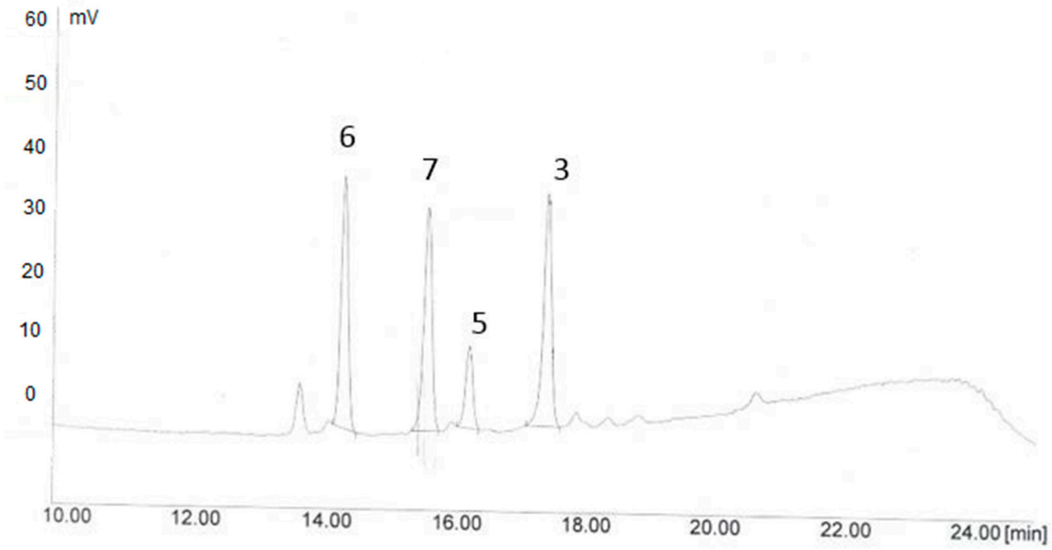

Figure S1. HPLC chromatogram of compounds **3**, **5**, **6**, **7**. Method: Jasco 880-PU (Jasco Europe, Cremella, Italy) pump equipped with a Jasco 875-UV/V detector; RP-18e (5 µm) column (Purosphere STAR, 100 mm × 3 mm, Merck, Germany; solvent system A: $MeCN/H_2O/HCO_2H$ (5/95/0.1 *v*/*v*/*v*) and B: $MeCN/HCO_2H$ (100/0.1 *v*/*v*). Gradient 0–1 min 30% B, 1–20 min 30%–80% B, 20–22 min 80% B, 22–24 min 30% B. Flow rate was 1.1 mL/min. The data were acquired at 285 nm. (Method B).

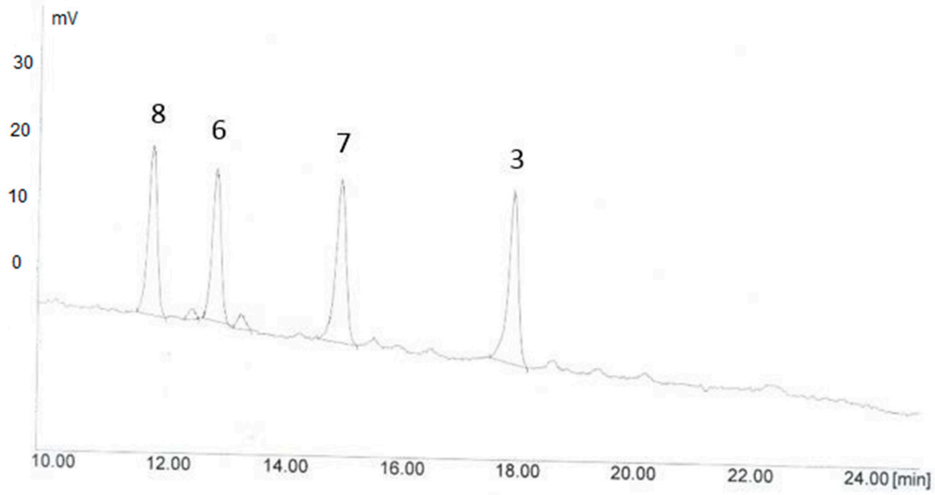

Figure S2. HPLC chromatogram of compounds **3**, **6**, **7**, **8**. Method: Jasco 880-PU (Jasco Europe, Cremella, Italy) pump equipped with a Jasco 875-UV/V detector; RP-18e (5 µm) column (Purosphere STAR, 100 mm × 3 mm, Merck, Germany); solvent system A: $MeCN/H_2O/HCO_2H$ (5/95/0.1, *v*/*v*/*v*) and B: $MeCN/HCO_2H$ (100/0.1, *v*/*v*). Gradient 0–1 min 40% B, 1–20 min 40%–70% B, 20–22 min 70% B, 22–24 min 40% B. Flow rate was 1.1 mL/min. The data were acquired at 285 nm. (Method B).

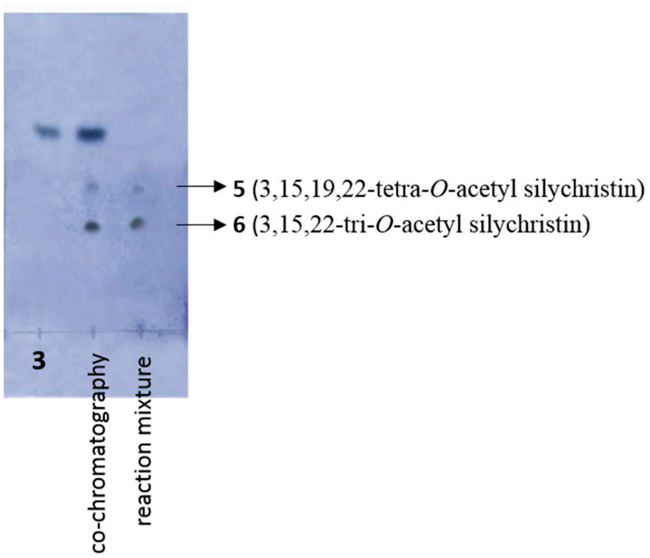

Figure S3. TLC (Thin layer chromatography) of the reaction mixture catalyzed by lipase PS after 100 h. Compound **3** (3,5,7,15,19,22-hexa-*O*-acetyl silychristin) was a starting material for the alcoholysis catalyzed by lipase PS. Co-chromatography was performed with **3** and reaction mixture. $CHCl_3$/toluene/acetone/HCO_2H, 12/2/2/0.1 was used as mobile phase. TLC plate was developed with Pancaldi reagent ($(NH_4)_6MoO_4$ 42 g, $Ce(SO_4)_2$ 2 g, H_2SO_4 62 mL, H_2O 1 L).

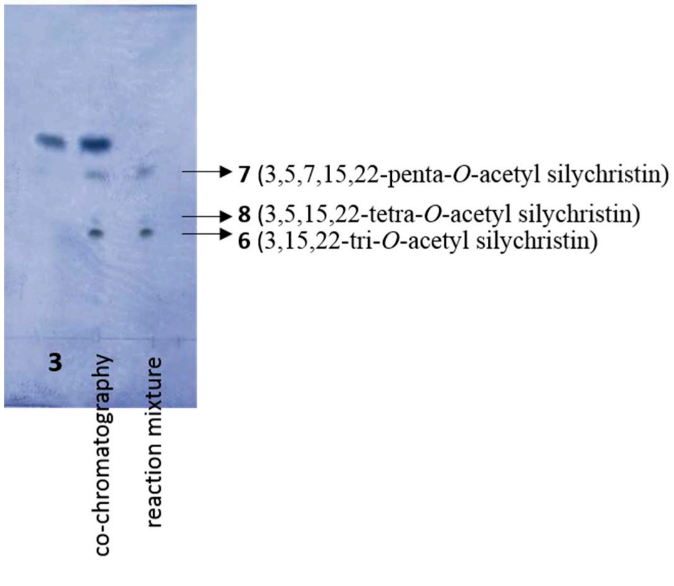

Figure S4. TLC of the reaction mixture catalyzed by Novozym 435 after 52 h. Compound **3** (3,5,7,15,19,22-hexa-*O*-acetyl silychristin) was a starting material for the alcoholysis catalyzed by Novozym 435. Co-chromatography was performed with **3** and reaction mixture. $CHCl_3$/toluene/acetone/HCO_2H 12/2/2/0.1 was used as mobile phase. TLC plate was developed with Pancaldi reagent ($(NH_4)_6MoO_4$ 42 g, $Ce(SO_4)_2$ 2 g, H_2SO_4 62 mL, H_2O 1 L).

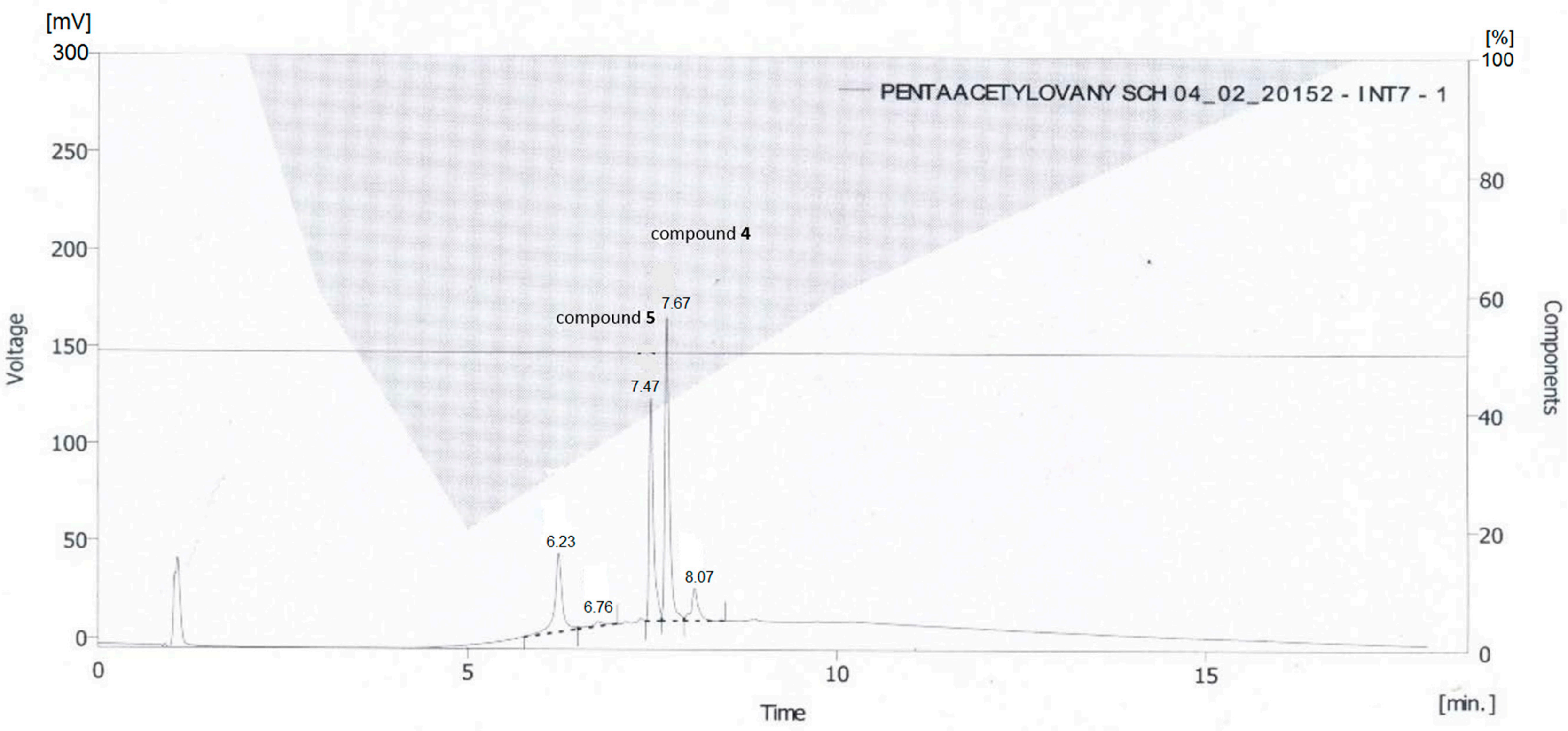

Figure S5. HPLC chromatogram of the inseparable mixture of two products 3,5,15,19,22-penta-*O*-acetyl-silychristin (**4**) and 3,15,19,22-tetra-*O*-acetyl silychristin (**5**). Twin Watrex (Prague, Czech Republic) Deltachrom SDS (030) pumps equipped with Thermo Separation Spectra 100 UV/V detector; RP-18e Chromolith ® SpeedRod (50 mm × 4.6 mm, Merck, Darmstadt, Germany); solvent system A: MeOH/H_2O/HCO_2H (20/80/0.1, *v*/*v*/*v*) and B: MeOH/HCO_2H (100/0.1, *v*/*v*). Gradient: 0–2 min 100% A, 2–3 min 100%–60% A, 3–5 min 60%–20 % A, 5–10 min 20%–60% A, 10–17 min 60%–100% A. (gradient depicted by the grey graph); flow rate 0.9 mL/min; signal at 285 nm was acquired. (Method C).

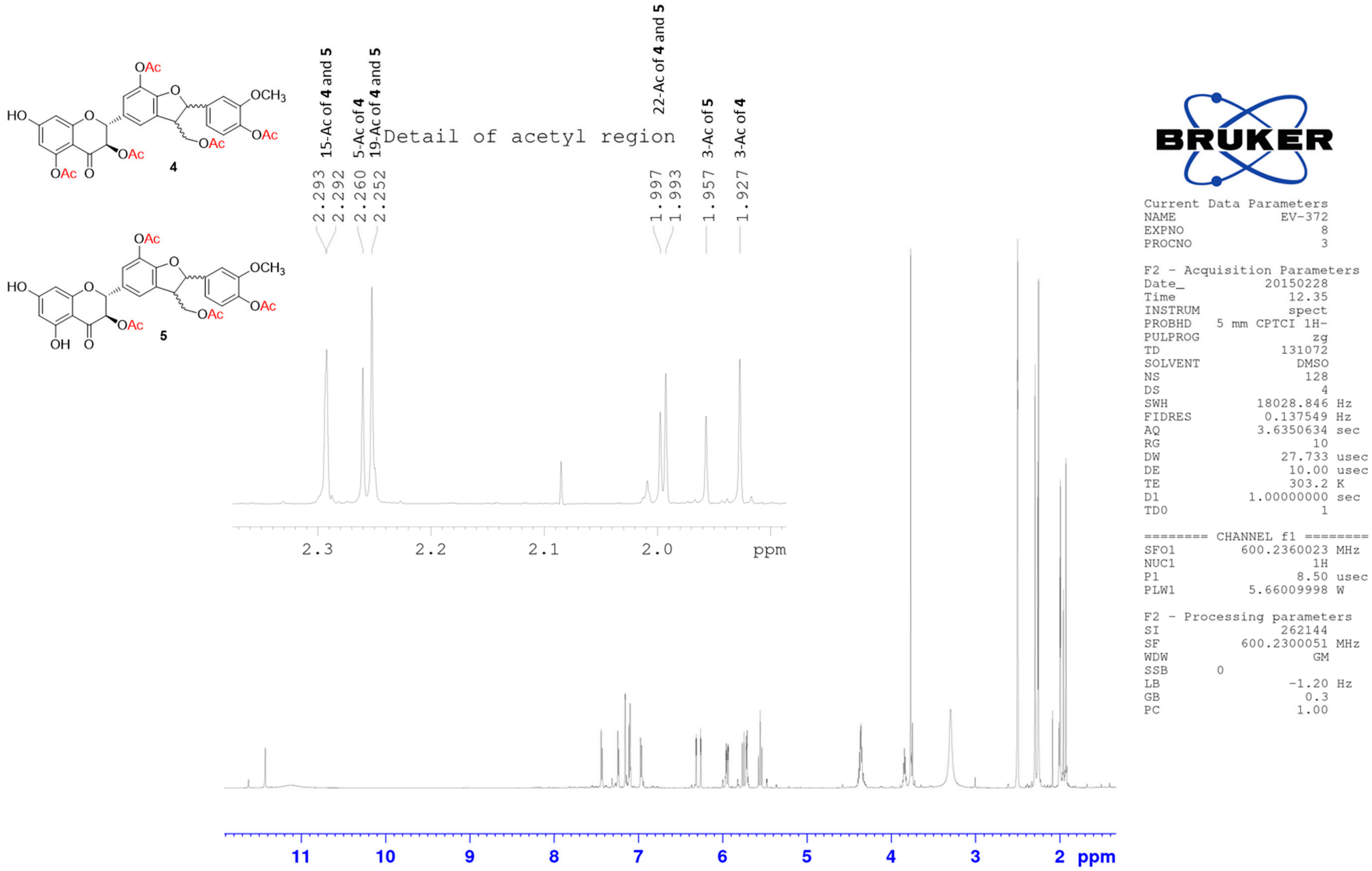

Figure S6. ^{1}H NMR spectrum of the mixture of compounds **4** and **5**. NMR spectrum was recorded on a Bruker Avance III 600 MHz spectrometer (600.23 MHz for ^{1}H 30 °C) in DMSO-d_6.

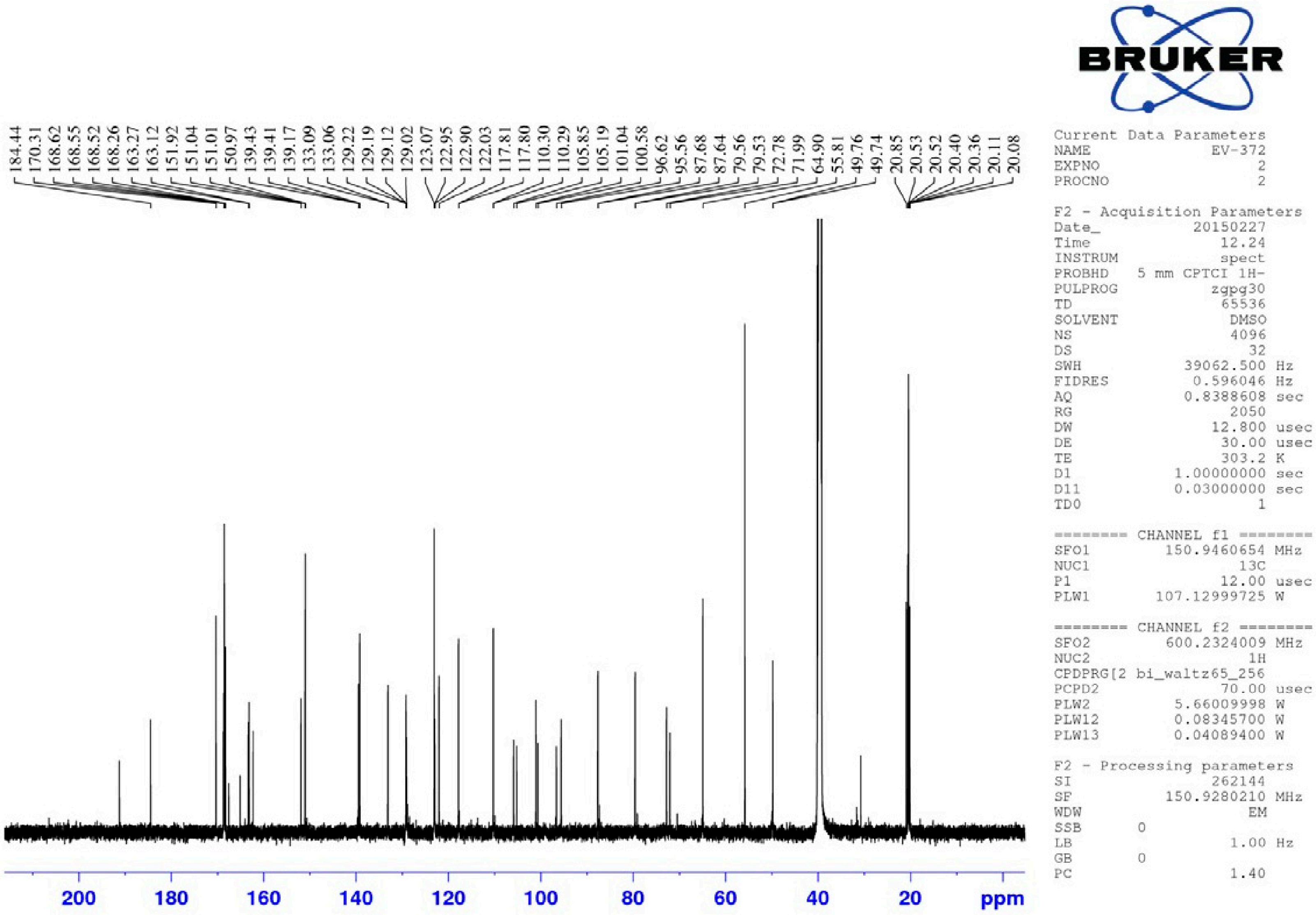

Figure S7. ^{13}C NMR spectrum of the mixture of compounds **4** and **5**. NMR spectrum was recorded on a Bruker Avance III 600 MHz spectrometer (150.93 MHz for ^{13}C at 30 °C) in DMSO-d_6.

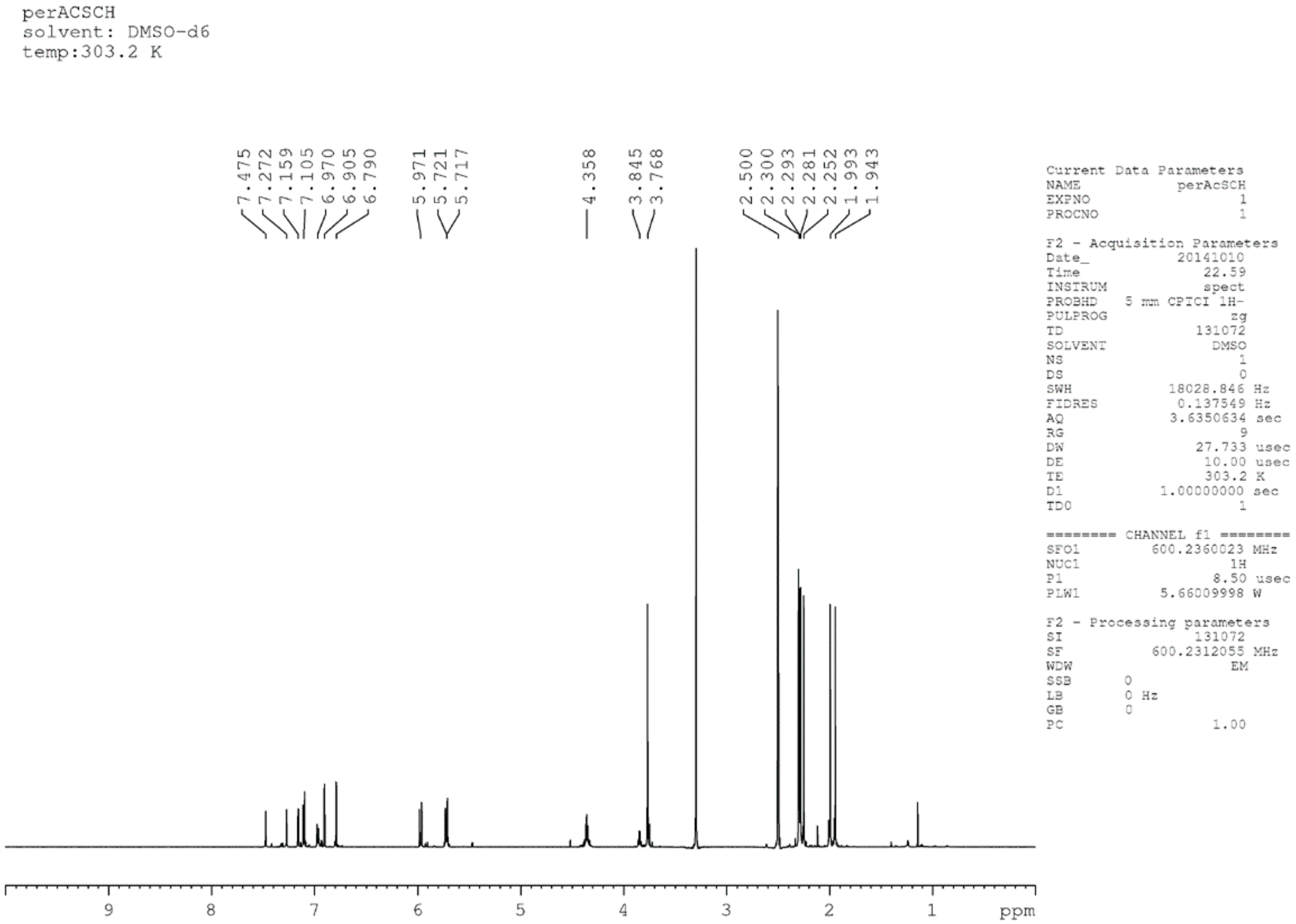

Figure S8. ^{1}H NMR spectrum of the compound **3**. NMR spectrum was recorded on a Bruker Avance III 600 MHz spectrometer (600.23 MHz for ^{1}H 30 °C) in DMSO-d_6.

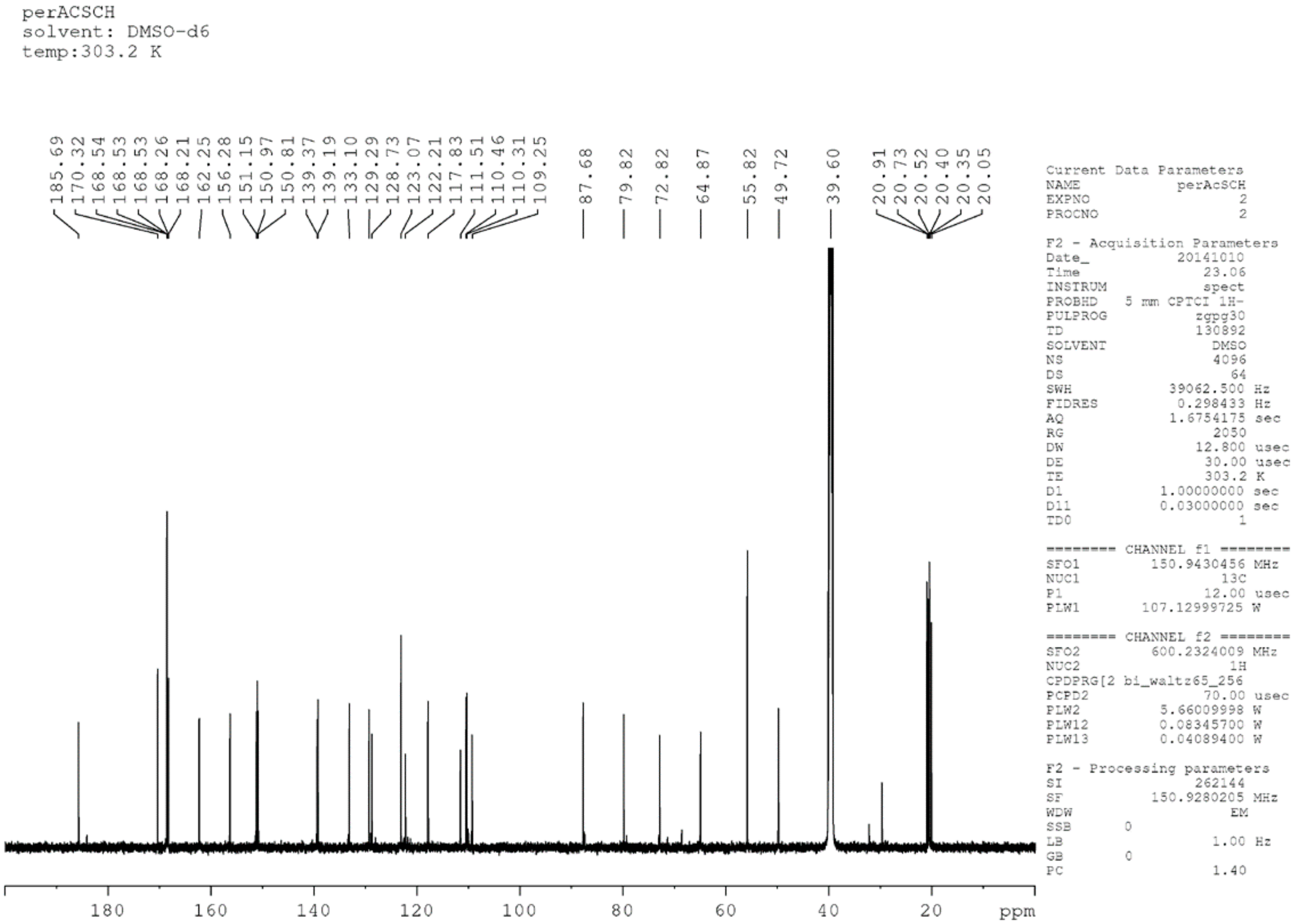

Figure S9. ^{13}C NMR spectrum of the compound **3**. NMR spectrum was recorded on a Bruker Avance III 600 MHz spectrometer (150.93 MHz for ^{13}C at 30 °C) in DMSO-d_6.

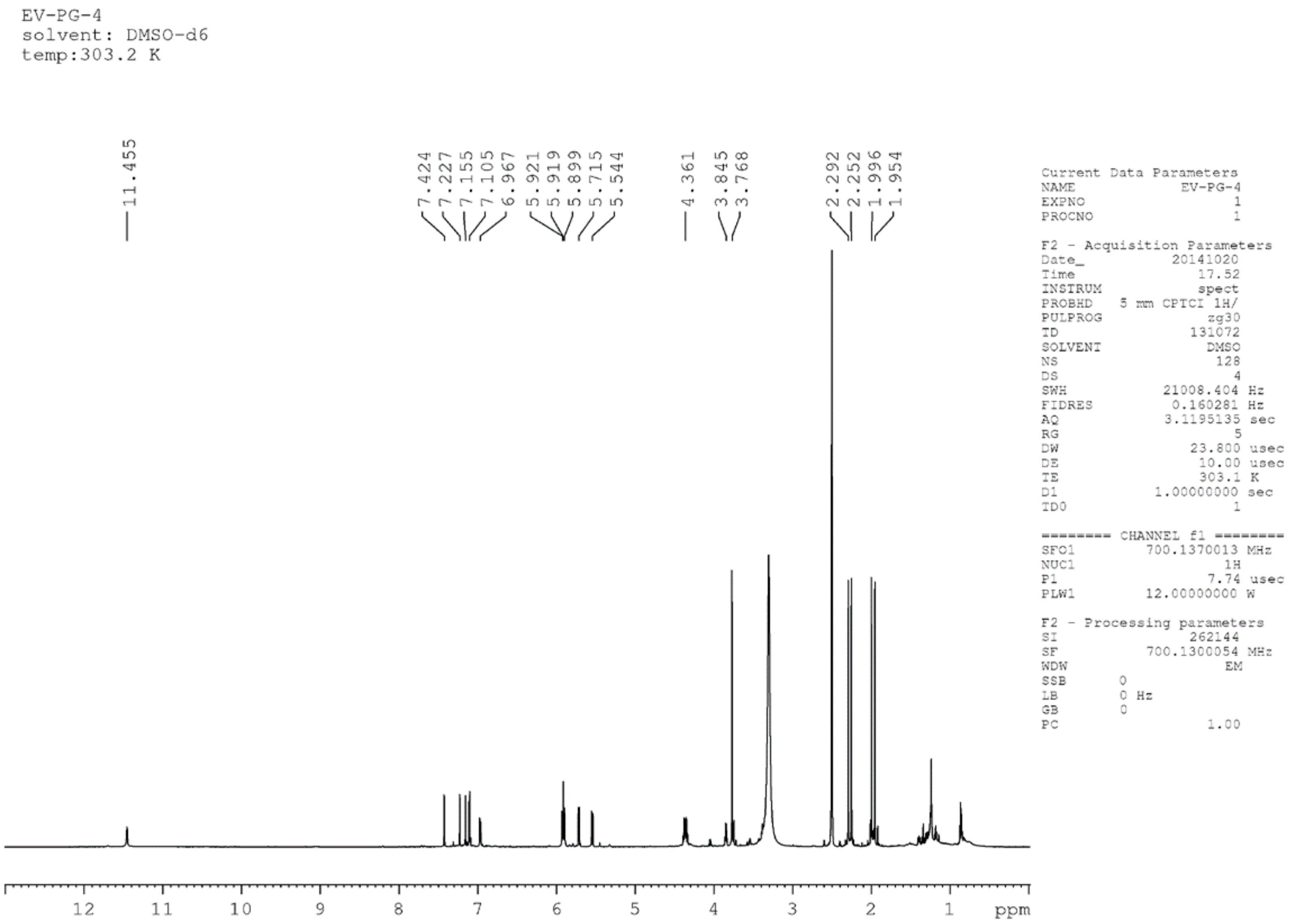

Figure S10. ^{1}H NMR spectrum of the compound **5**. NMR spectrum was recorded on a Bruker Avance III 700 MHz spectrometer (700.13 MHz for ^{1}H 30 °C) in DMSO-d_6.

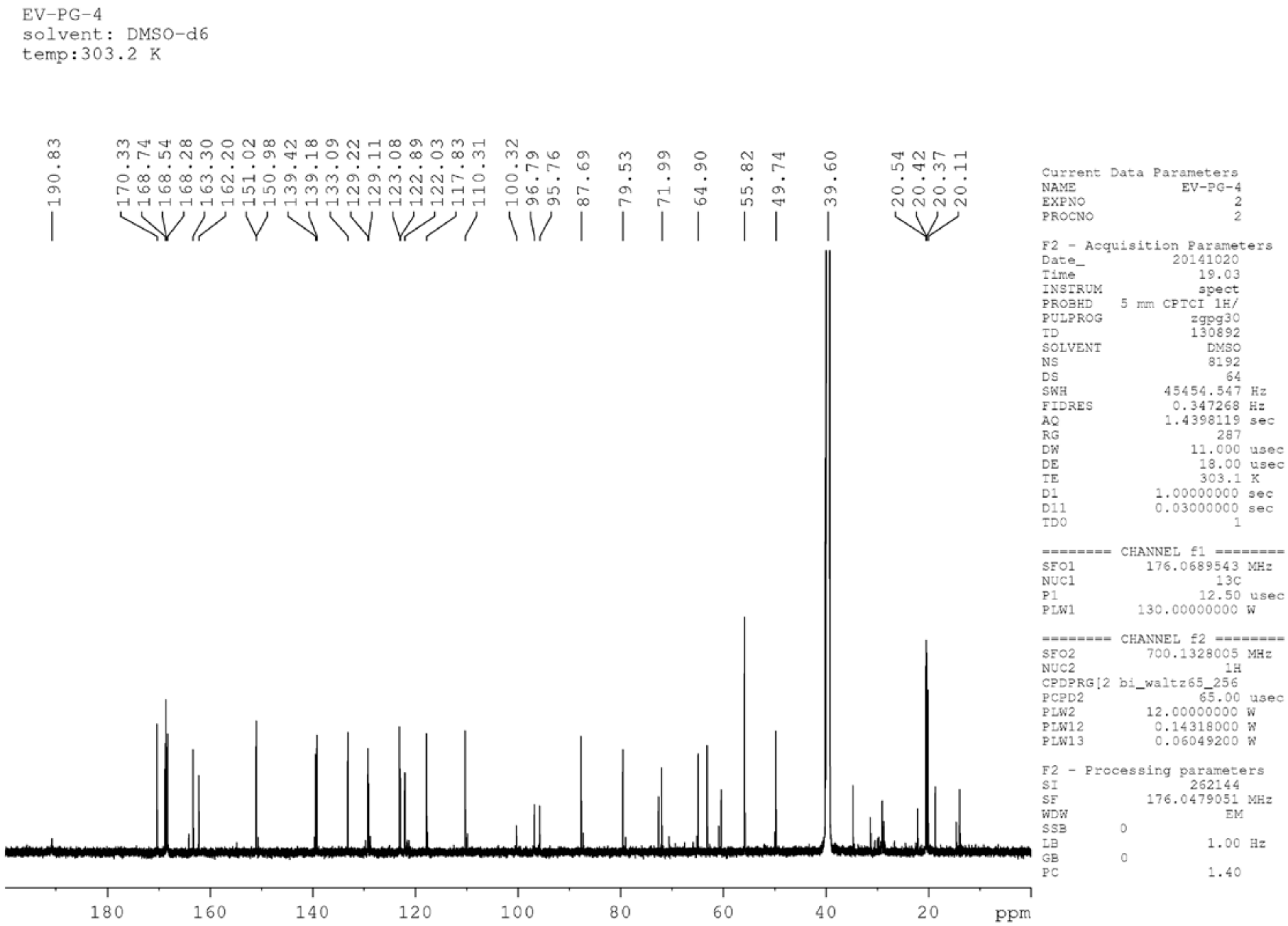

Figure S11. ^{13}C NMR spectrum of the compound **5**. NMR spectrum was recorded on a Bruker Avance III 700 MHz spectrometer (176.07 MHz for ^{13}C at 30 °C) in DMSO-d_6.

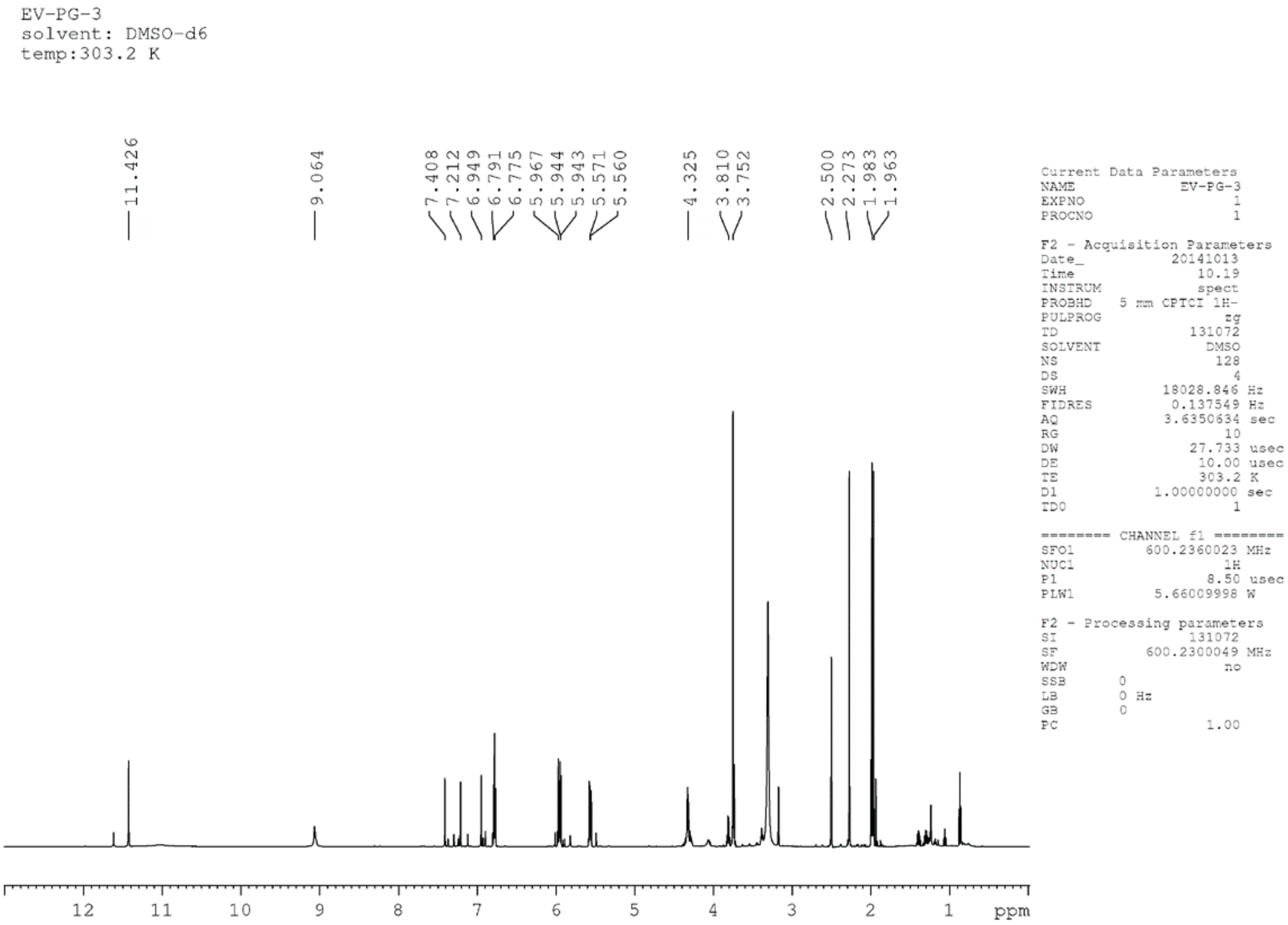

Figure S12. ^{1}H NMR spectrum of the compound **6**. NMR spectrum was recorded on a Bruker Avance III 600 MHz spectrometer (600.23 MHz for ^{1}H 30 °C) in DMSO-d_6.

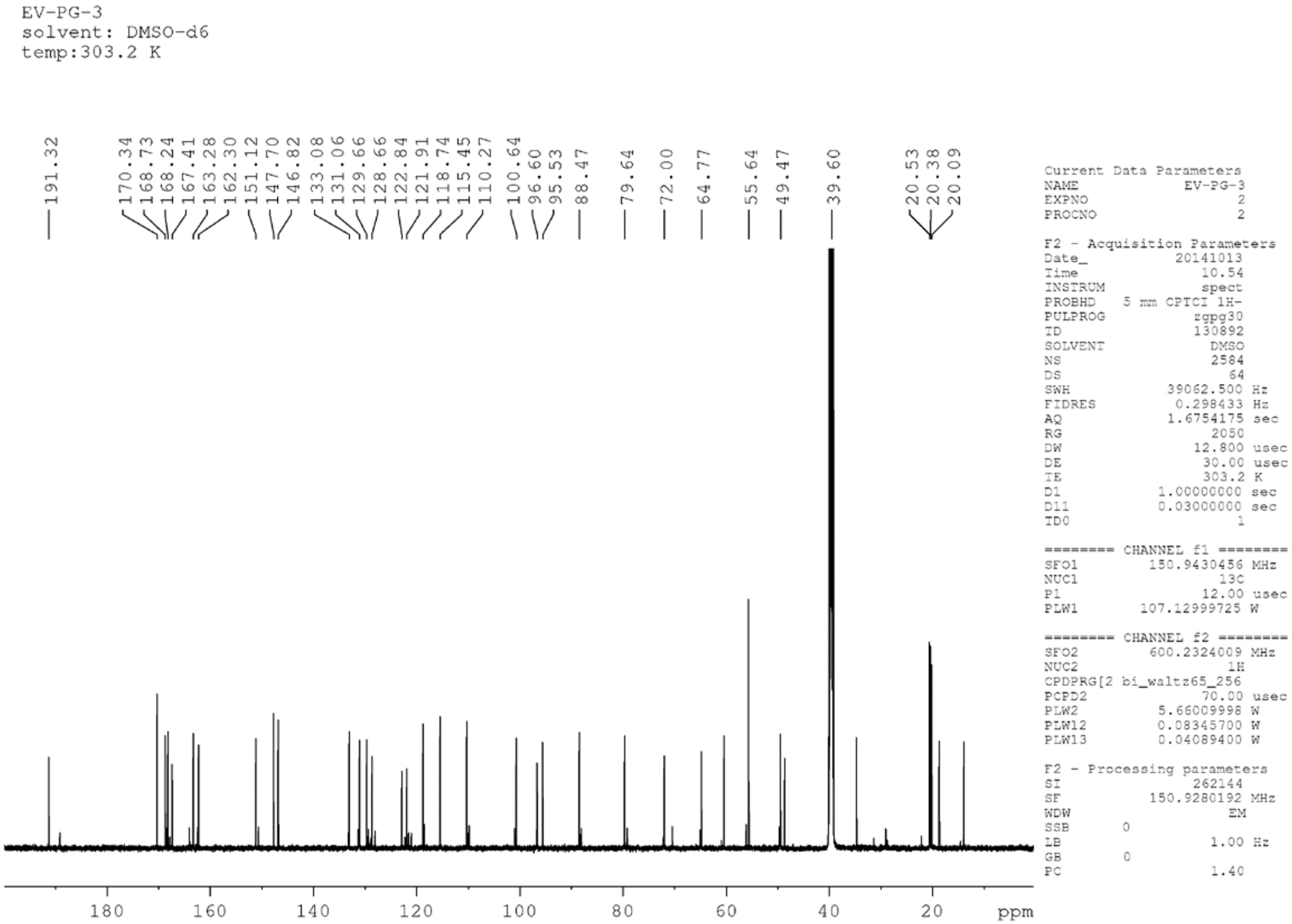

Figure S13. ^{13}C NMR spectrum of the compound **6**. NMR spectrum was recorded on a Bruker Avance III 600 MHz spectrometer (150.94 MHz for ^{13}C at 30 °C) in DMSO-d_6.

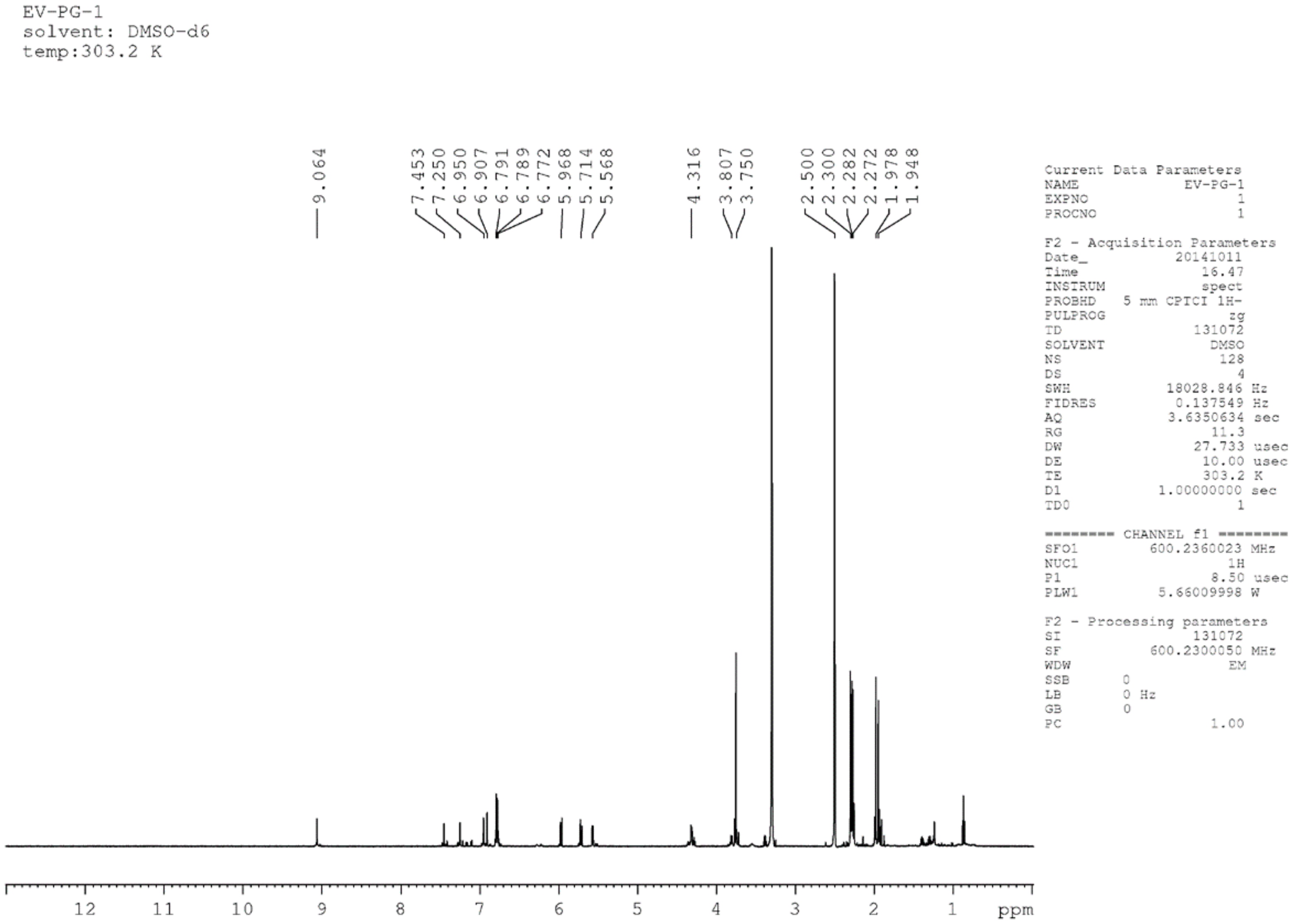

Figure S14. ^{1}H NMR spectrum of the compound **7**. NMR spectrum was recorded on a Bruker Avance III 600 MHz spectrometer (600.23 MHz for ^{1}H 30 °C) in DMSO-d_6.

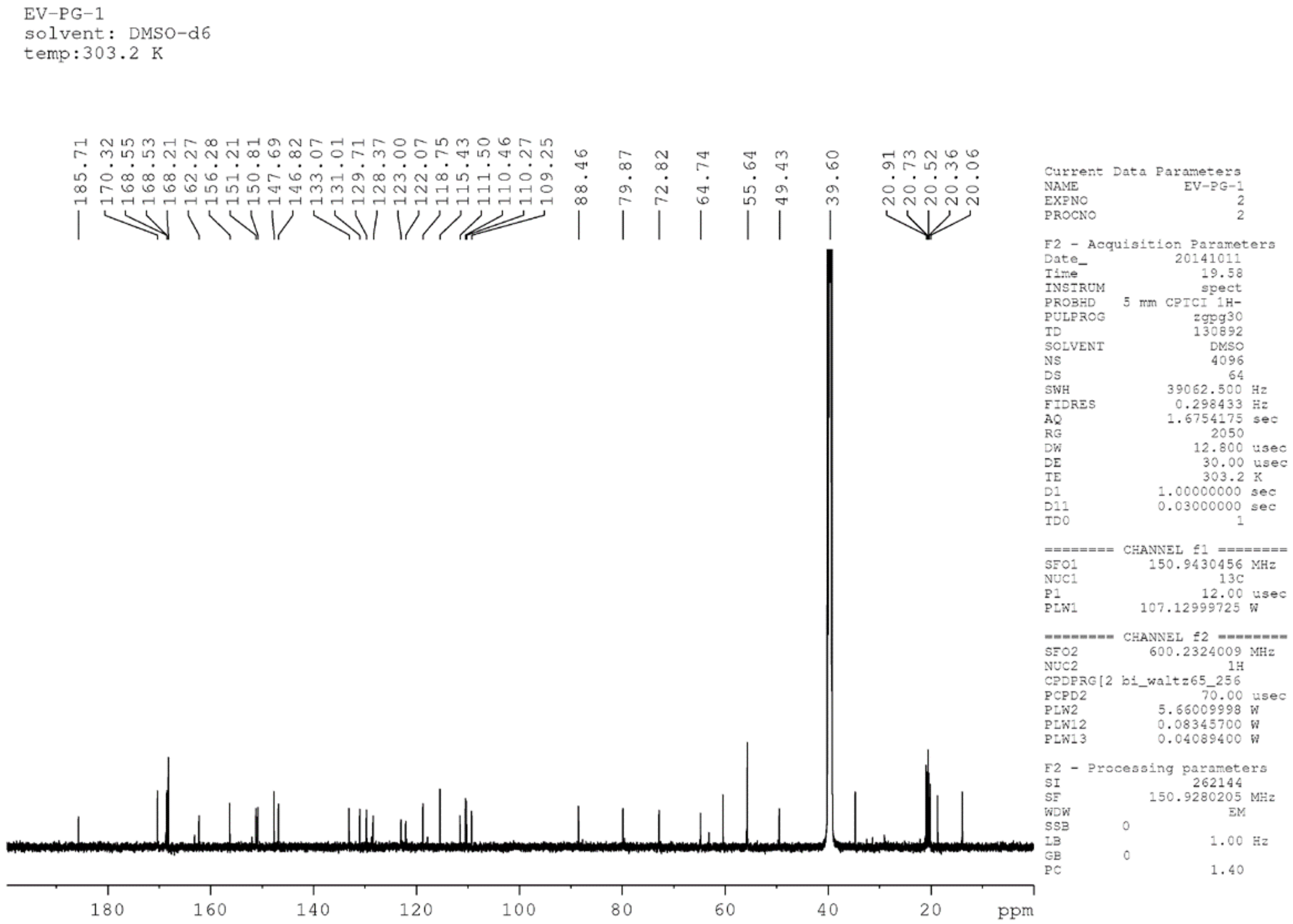

Figure S15. ^{13}C NMR spectrum of the compound **7**. NMR spectrum was recorded on a Bruker Avance III 600 MHz spectrometer (150.94 MHz for ^{13}C at 30 °C) in DMSO-d_6.

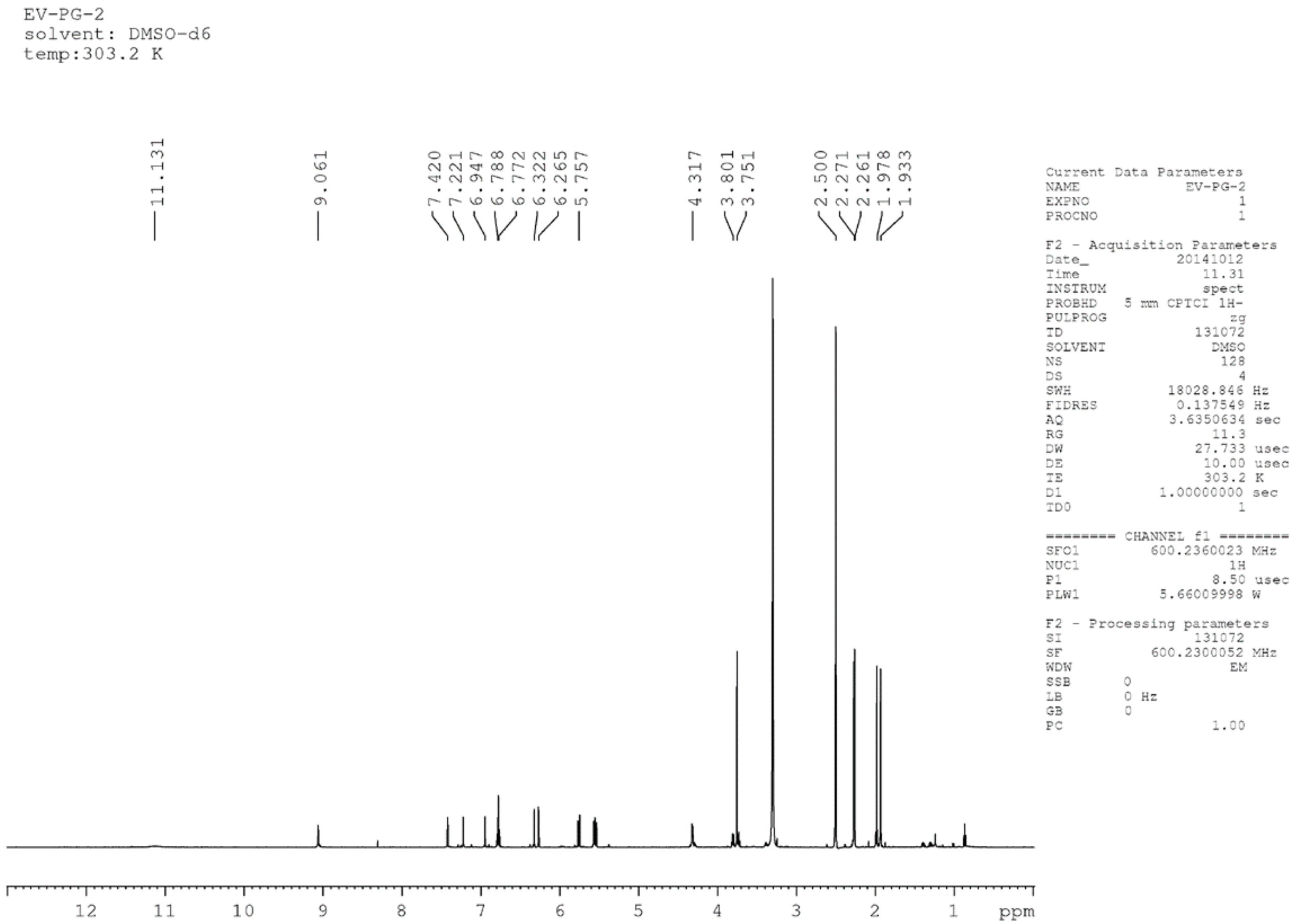

Figure S16. ^{1}H NMR spectrum of the compound **8**. NMR spectrum was recorded on a Bruker Avance III 600 MHz spectrometer (600.23 MHz for ^{1}H 30 °C) in DMSO-d_6.

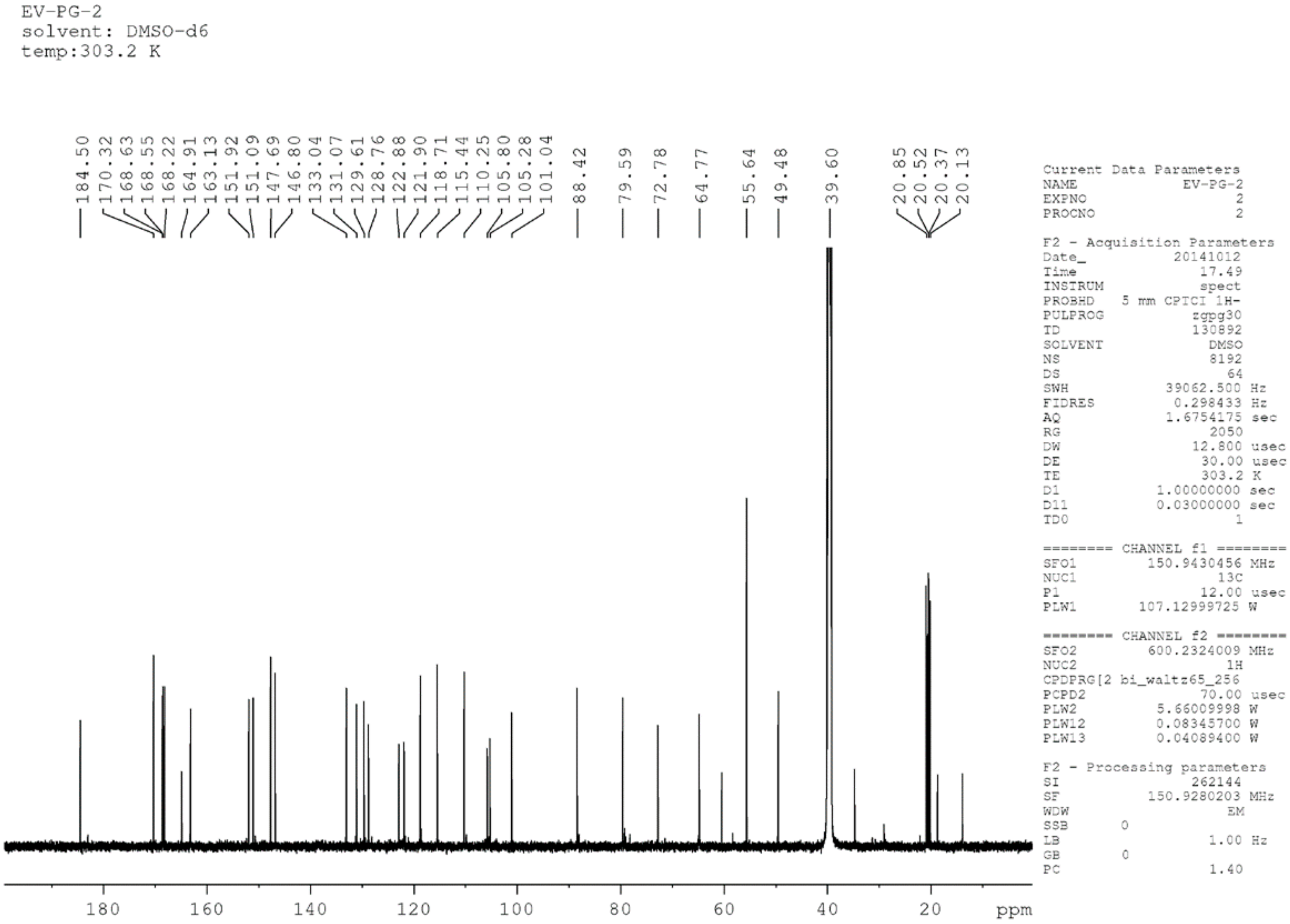

Figure S17. ^{13}C NMR spectrum of the compound **8**. NMR spectrum was recorded on a Bruker Avance III 600 MHz spectrometer (150.94 MHz for ^{13}C at 30 °C) in DMSO-d_6.
